# Mercapto-NSAIDs generate a non-steroidal anti-inflammatory drug (NSAID) and hydrogen sulfide†

**DOI:** 10.1039/d4sc08525f

**Published:** 2025-02-05

**Authors:** Simran M. Gupta, Pratiksha S. Mohite, Harinath Chakrapani

**Affiliations:** a Department of Chemistry, Indian Institute of Science Education and Research Pune Pune 411 008 Maharashtra India harinath@iiserpune.ac.in

## Abstract

Non-steroidal anti-inflammatory drugs (NSAIDs) are among the frontline treatments for inflammation and pain. Hydrogen sulfide (H_2_S) and related persulfide (RS-SH) are important mediators of antioxidant response and protect cells from oxidative stress. Hybrids of these pharmacological agents have shown promise in clinical trials and are superior to the parent NSAID. Here, we report a new class of NSAID–H_2_S hybrids, where a strategic placement of a sulfhydryl group adjacent to a carbonyl of a NSAID facilitates the enzymatic generation of H_2_S. We show that α-mercapto-nabumetone, a derivative of the clinical drug nabumetone, is a substrate for 3-mercaptopyruvate sulfurtransferase (3-MST), an enzyme involved in H_2_S biosynthesis. The key step of 3-MST catalysis is the cleavage of a C–S bond adjacent to a carbonyl group, which generates an enolate and 3-MST persulfide, which in turn is cleaved under reducing conditions to generate H_2_S. Guided by a molecular docking study with 3-MST, we prepared two mercapto-nabumetone derivatives, protected as their thioacetates. In the presence of 3-MST, both mercapto-nabumetone derivatives generated H_2_S and the NSAID in a nearly quantitative yield, produced glutathione persulfide (GS-SH), an important mediator of cellular antioxidant response, and permeated cells to generate H_2_S. Lastly, to gain insights into the scope of this strategy, we prepared mercapto-NSAID derivatives containing a carboxylic acid. We found that the propensity to generate H_2_S depended on the nature of the enol that is produced during the transformation of the mercapto-NSAID into the parent NSAID. This offers new insights into 3-MST catalysis and how reaction outcomes can be modulated by the keto–enol equilibrium. Taken together, the atom economical transformation of a clinical NSAID with one strategically placed sulfhydryl group to generate H_2_S presents new opportunities to enhance the properties of NSAIDs through participation in endogenous H_2_S biosynthesis.

## Abstract 2

A new class of mercapto-NSAIDs that generate persulfide, H_2_S, and the NSAID is reported.

## Graphical abstract

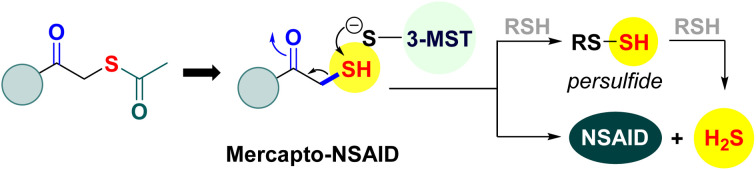

## Introduction

**Fig. 1:**
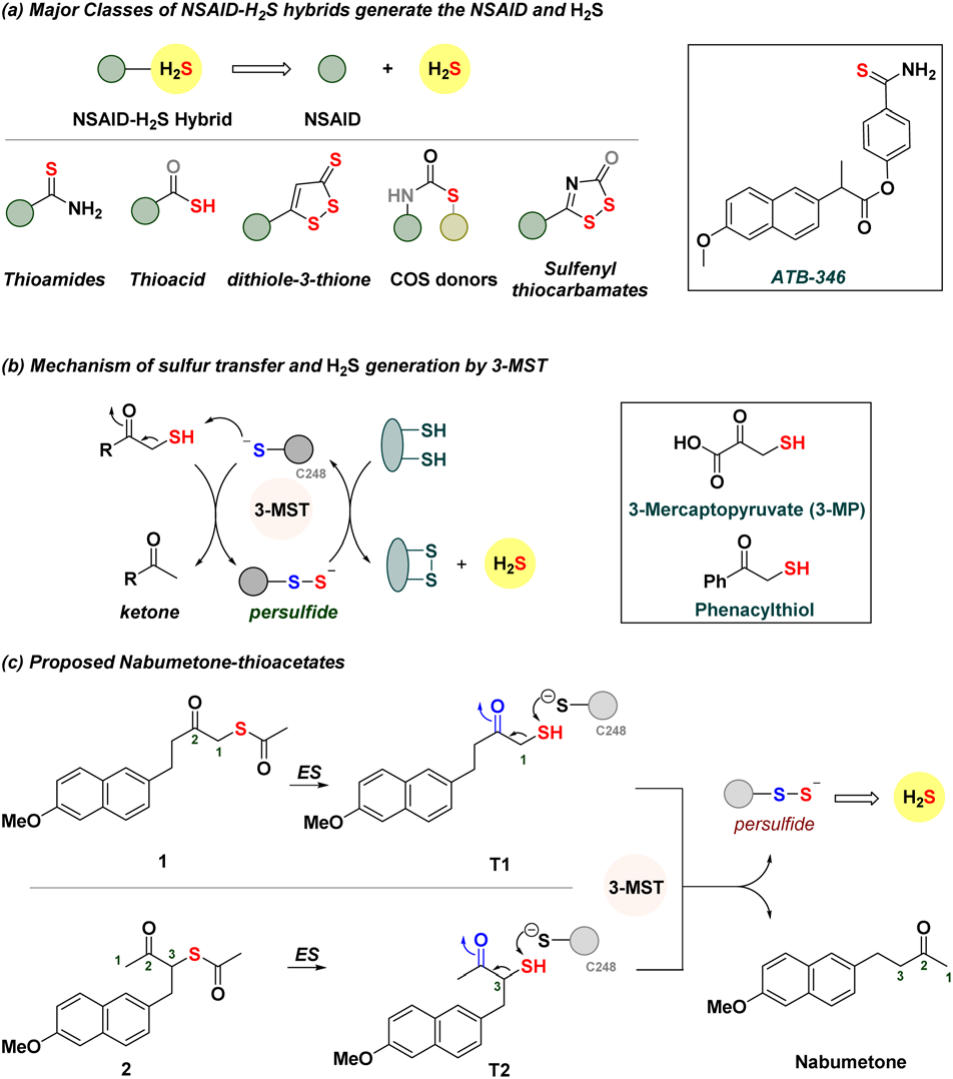
(a) Major classes of NSAID–H_2_S hybrids. These compounds dissociate to produce the NSAID and H_2_S. (b) Mechanism of persulfide and H_2_S generation by 3-MST. In the box are 3-mercaptopyruvate and phenacylthiol. (c) Proposed NSAID hybrids should be cleaved by esterase (ES) to generate the thiol, which reacts with 3-MST to generate a persulfide and the NSAID nabumetone.

Hydrogen sulfide (H_2_S) and related sulfane sulfur species are components of a cell's antioxidant machinery, and therapeutic strategies to use such species to promote antioxidant response to stress are in development.^1–8^ Exogenous sources of H_2_S, known as H_2_S donors, are small molecules that dissociate to generate H_2_S.^4–9^ From inorganic sources to compounds that require enzymatic activation, many classes of H_2_S donors are known, and some have promising antioxidant properties.^8–12^ Non-steroidal anti-inflammatory drugs (NSAIDs) are the mainstay treatment of pain and inflammation.^13–15^ These drugs have some limitations that have been shown to be mitigated by H_2_S.^16–18^ Hence, NSAID–H_2_S hybrids (Fig. 1a) are in development, and these hybrids have distinct pharmacological effects and appear to synergize to improve the overall efficacy of the NSAID. For example, the thioamide ATB-346 is a promising candidate for the treatment of post-operative pain, inflammation and osteoarthritis.^19^ This compound undergoes metabolism to generate H_2_S and the NSAID and has been found to reduce incidences of ulcers in patients when compared with the parent NSAID. A number of other hybrids have been similarly developed, with combinations of NSAIDs with varying degrees of success, and typically involve the addition of bulky groups and linkers.^19–24^ Some generate H_2_S spontaneously, while others are activated by thiols, enzymes, or reactive oxygen species (ROS).^25–31^

We considered using 3-mercaptopyruvate sulfurtransferase (3-MST), an endogenous enzyme that generates persulfide and H_2_S (Fig. 1b).^32–35^ The natural substrate is 3-mercaptopyruvate (3-MP), which interacts with the active site cysteine (C248) of 3-MST.^36^ Transfer of the sulfhydryl group of 3-MP by the cleavage of a C–S bond to 3-MST produces a persulfide, which, under reductive conditions, generates H_2_S.^37^ We recently developed phenacylthiol as an artificial substrate for this enzyme and found that this α-mercaptoketone produced H_2_S in the presence of 3-MST (Fig. 1b).^38,39^ Taking a cue from this result, we considered the clinically used ketone nabumetone (Fig. 1c)^40^ as a test case for insertion of a sulfhydryl group adjacent to the ketone, at either the C-1 or the C-3 position (structures T1 and T2, Fig. 1c). Nabumetone is among the widely used NSAIDs for the treatment of pain and inflammation as well as conditions such as osteoarthritis and rheumatoid arthritis.^41–43^ If these mercapto-NSAIDs are substrates for 3-MST, they are expected to regenerate the NSAID, nabumetone, and produce H_2_S. Since thiols themselves are prone to oxidation^44^ and are difficult to store for extended durations, we designed two thioacetates 1 and 2; hydrolysis by esterase (ES) should generate T1 and T2, respectively.^45^ Here, we report the synthesis and evaluation of α-mercapto-nabumetone, a new class of NSAID–H_2_S hybrids.

## Results and discussion

### Molecular docking study

**Fig. 2:**
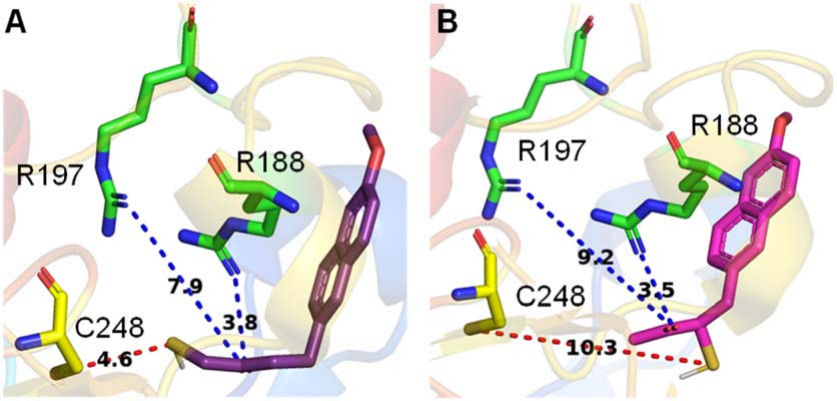
Lowest energy conformations found by molecular docking studies with human 3-MST of (A) T1 and (B) T2. Both T1 and T2 show comparable docking energy, *i.e.*, −5.5 kcal mol^−1^ and −5.3 kcal mol^−1^, respectively. C is the cysteine residue and R represents arginine. The distance between the sulfur of T1 and the sulfur of active residue C248 is 4.6 Å, whereas for T2 it is 10.3 Å.

The active site of the enzyme 3-mercaptopyruvate sulfurtransferase (PDB ID: 4JGT) was identified based on previous reports.^46,47^ The residues that are involved in binding and catalysis of 3-MP are C248, R188, and R197 (Fig. 2). Using standard molecular docking protocols, the binding of thiols T1 and T2 was first analysed. The lowest energy binding conformation of T1 was found to be favorable (−5.5 kcal mol^−1^). An important parameter that facilitates turnover and product formation is the S⋯S distance between the cysteine sulfur and T1.^38,46^ This distance was found to be 4.6 Å, which from our previous studies supports a favorable binding interaction (Fig. 2A).^38^ The carbonyl group was also found to be proximal to the two arginine residues, and this interaction has been previously considered important for the stabilization of the incipient enolate.^48^

Similarly, when T2 was analysed, although the binding energy was favorable (−5.3 kcal mol^−1^), the S⋯S distance between the cysteine and T2 was somewhat longer (10.3 Å), suggesting a less favorable interaction that eventually leads to persulfide formation (Fig. 2B). Hence, both thiols are predicted to be substrates for 3-MST, with T1 being more favourable for binding and positioning in the active site, as compared with T2 (Table S1, see the ESI†).

### Synthesis

**Scheme 1:**
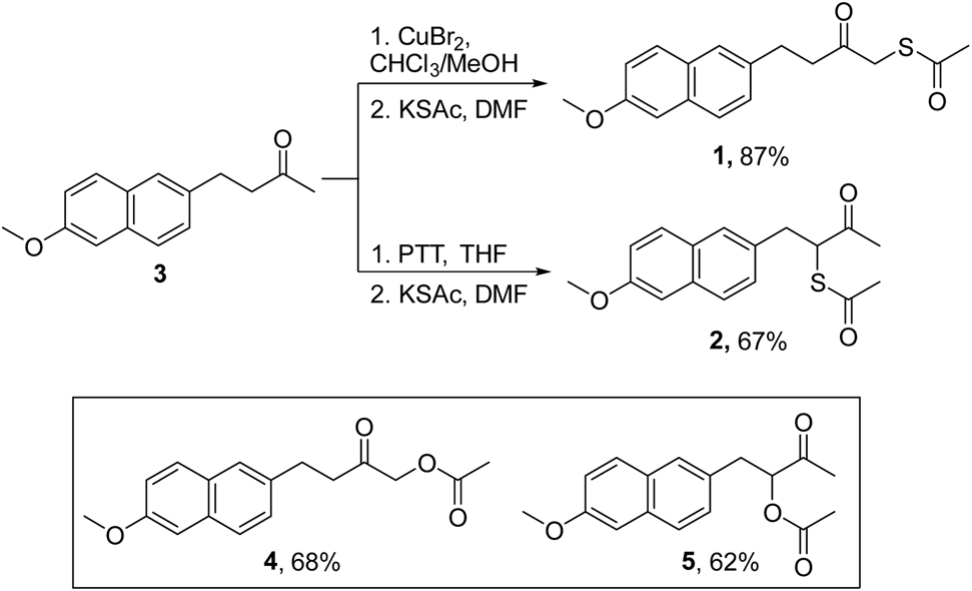
Synthesis of mercapto-nabumetone derivatives 1 and 2; inset: structures of negative controls 4 and 5 (Schemes S1, see the ESI†).

Nabumetone (3) was first converted to 1-bromo-nabumetone using copper bromide (CuBr_2_),^49^ and the yield was found to be 45% (Scheme 1). The bromide was subsequently converted to 1 using potassium thioacetate with a yield of 87% (Scheme 1). Similarly, to synthesize 2, 3-bromo-nabumetone was prepared using phenyltrimethyl ammonium tribromide (PTT)^50^ as the brominating agent in 72% yield (Scheme 1). The reaction of this bromide with potassium thioacetate gave 2 in 67% yield. The oxygen versions 4 and 5 were synthesized as negative controls in 68% and 62% yields, respectively (Schemes 1 and S1, see the ESI†).

### Stability studies

First, to determine whether 1 was stable in pH 7.4 buffer, HPLC analysis was carried out. The thioacetate was found to be stable over 90 min (Fig. S1A, see the ESI†). In the presence of esterase, as expected, the thioacetate produced T1 (Fig. S2A, see the ESI†) along with a disulfide, due to aerial oxidation. Independently, T1 was trapped by mono-bromobimane (mBBr) as a bimane adduct (MALDI analysis, Scheme S2, Fig. S3, see the ESI†). In the presence of ES and dithiothreitol (DTT), T1 was produced and remained stable (Fig. S4A, see the ESI†). In the case of 2, under similar conditions, only 54% of the thioacetate remained in 90 min in pH 7.4 buffer (Fig. S1, see the ESI†). The formation of T2 as the major product was seen, indicating that the thioester of 2 was cleaved faster than 1. The origin of this increased lability is not clear but the C=O stretching frequency for the thioacetate for 2 was higher than for 1 (2, 1695 cm^−1^*vs.*1, 1689 cm^−1^) suggesting an increased reactivity. No further reaction of the thiol with DTT was seen, suggesting that both T1 and T2, once formed, largely remained intact under reducing conditions (Fig. S4B, see the ESI†). Derivatives 1 and 2 were also found to be stable at acidic pH for 60 min (pH 2 and 6.8, Fig. S5 and S6, see the ESI†). Hence, the thioacetate can be converted to the thiol in the presence of biothiols as well as ES; the thiols T1 and T2 were themselves quite stable at acidic and neutral pH as well as in the presence of biothiols at pH 7.4.

### Catalysis by 3-MST to generate nabumetone

**Fig. 3:**
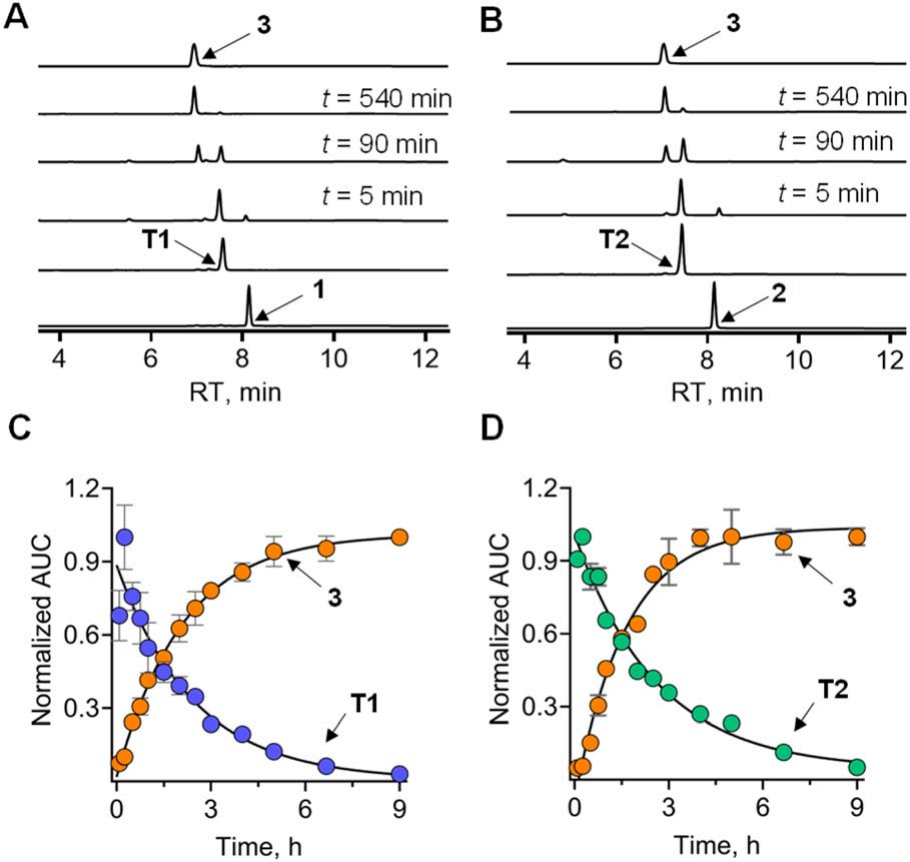
(A and B) Representative HPLC traces of 1 or 2 (100 μM) in the presence of ES (1 U mL^−1^), 3-MST (1 μM), and DTT (1 mM), showing the formation of nabumetone 3 from *t* = 0 to *t* = 540 min. T1 and T2 are traces where 1 or 2 has been treated with ES and DTT; (C and D): curve fitting gave an apparent rate constant for the decomposition of T1 and T2 with a *k*_T1_ of 0.38 h^−1^ and a *k*_T2_ of 0.4 h^−1^, and the formation of nabumetone with 0.57 h^−1^ and 0.6 h^−1^, respectively. All data are represented as mean ± SD (*n* = 2). SD is the standard deviation.

Next, to determine whether the thiol could generate persulfide through sulfur transfer, a reaction of 1 in the presence of ES, 3-MST, and DTT was performed (Scheme S3 and Fig. S7A, see the ESI†). Under these conditions, it is expected that 1 is converted to T1, which in turn would be turned over by 3-MST to produce 3-MST persulfide and nabumetone (Fig. 3A). DTT is expected to cleave 3-MST persulfide to regenerate the active catalyst. In the presence of ES, 3-MST, and DTT, a nearly complete disappearance of 1 was seen in 30 min. The thiol T1 was formed and was gradually converted to nabumetone over several hours (Fig. 3C). The observed rate constant for the disappearance of T1 (*k*_T1_) was 0.38 h^−1^ and for the formation of nabumetone (*k*_3_) was 0.57 h^−1^. The yield of nabumetone was nearly quantitative, supporting a clean and efficient conversion of 1 to produce the NSAID (Fig. S7A, see the ESI†). The hybrid 2 also produced T2, which disappeared (*k*_T2_ = 0.4 h^−1^) to produce nabumetone as the exclusive product, again in a nearly quantitative yield at a rate of *k*_3_ = 0.6 h^−1^ (Fig. 3B, D, and S7B, see the ESI†). No significant difference in the rates of nabumetone formation in the aforementioned reactions was seen. The disappearance of thiols also occurred at a comparable rate. Both thiols are equally good substrates for 3-MST, and it appears that the barrier for conversion of the most favorable but high energy conformation of T2 to a less favorable productive conformation is low.

### Sulfur transfer and sulfane sulfur generation

**Fig. 4:**
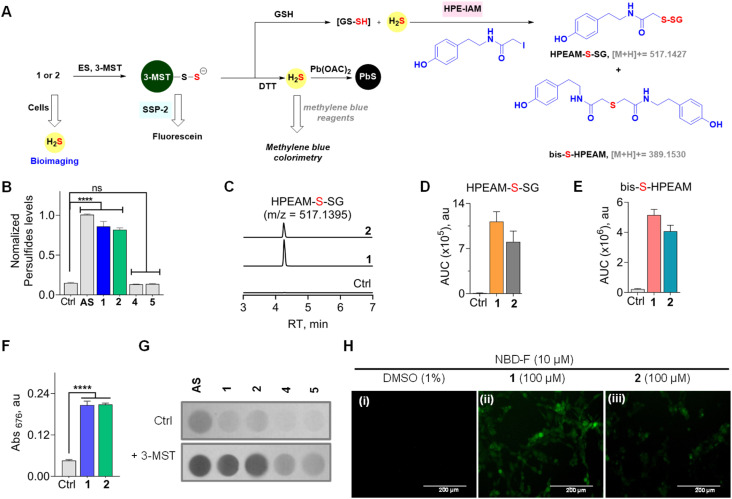
(A) Compounds 1 or 2 in the presence of ES and 3-MST should produce 3-MST persulfide, and when glutathione is added, the formation of glutathione persulfide (GS-SH) and H_2_S is expected. The intermediates of the above are independently detected by various assays. (B) An SSP-2-based fluorescence assay for the detection of persulfide: compounds (100 μM) were incubated with 3-MST (15 μM), ES (1 U mL^−1^), and SSP-2 (50 μM). Ctrl represents 3-MST only. *λ*_ex_ = 482 nm and *λ*_em_ = 518 nm. (C) LC-MS/MS analyses for persulfide and H_2_S: extracted ion chromatogram of the HPEAM-S-SG adduct (expected *m*/*z* = 517.1427; observed *m*/*z* = 517.1395) formed upon incubation of 1 or 2 with 3-MST (25 μM) in the presence of ES (1 U mL^−1^), followed by sequential addition of the thiol acceptor (GSH; 400 μM) and electrophilic trapping reagent (HPE-IAM; 1 mM). The formation of HPEAM-S-SG (GSSH formation) and bis-S-HPEAM (H_2_S formation) was monitored, and these data are available in (D) and (E). Ctrl represents only 3-MST under standard conditions. (D) The area under the curve (AUC) for the peak corresponds to HPEAM-S-SG. (E) The area under the curve (AUC) for the peak corresponds to the bis-S-HPEAM adduct (expected *m*/*z* = 389.1530; observed *m*/*z* = 389.1516). (F) H_2_S detection using methylene blue assay: 1 or 2 (100 μM) was incubated with 3-MST (1 μM), ES (1 U mL^−1^), and DTT (10 mM). Absorbance at 676 nm is a measure of H_2_S generated. Incubation time is 120 min. See ESI Fig. S9† for time course and kinetics analysis. (G) H_2_S detection using lead acetate assay: 1 or 2 (100 μM) was incubated in the presence of 3-MST (1 μM), ES (1 U mL^−1^), and DTT (10 mM). Ctrl represents the absence of 3-MST. (H) Representative images of MEF cells. The H_2_S-sensitive fluorogenic probe NBD-fluorescein (10 μM) was cotreated with (i) DMSO (1%); (ii) 1, 100 μM; (iii) 2, 100 μM for 1 h, followed by imaging using a green fluorescent protein filter (GFP). The scale bar is 200 μm. All data are represented as mean ± SD (*n* = 3). Statistical analysis carried out in (B) and (F): Student's two-tailed unpaired parametric *t*-test was used to determine significance: ****p < 0.0001 and ns = non-significant with respect to 3-MST only.

Next, two independent assays to assess sulfur transfer and persulfide generation were carried out (Fig. 4A). An assay based on the persulfide-sensitive fluorogenic probe SSP-2 was next used to detect 3-MST persulfide during catalysis (Scheme S4, see the ESI†).^51^ As expected, AS in the presence of ES and 3-MST gave a signal in this assay (Fig. 4B). In the absence of 3-MST, no significant signal was observed, supporting the need for catalysis by 3-MST to generate persulfide. Under similar conditions, 1 or 2 alone showed no detectable increase in the fluorescence signal (Fig. S8, see the ESI†). In the presence of ES and 3-MST, a significant enhancement of fluorescence, comparable with AS, was recorded. When this reaction mixture was co-treated with DTT, a nearly complete abrogation of fluorescence that is consistent with the cleavage of the 3-MST persulfide was observed (Fig. S8, see the ESI†). The oxygen versions 4 and 5 did not show any fluorescent signal (Fig. 4B).

Since 3-MST has been reported to persulfidate low molecular weight thiols, which could contribute to antioxidant response, we next tested if GS-SH, the persulfide of glutathione (GSH), could be formed under standard reaction conditions.^37,52–54^ Compound 1 or 2 was treated with β-(4-hydroxyphenyl)ethyl iodoacetamide (HPE-IAM) (Scheme S5, see the ESI†); this electrophile reacts with H_2_S and persulfides to produce distinct adducts, which can be analysed by LC-MS/MS (Fig. 4A).^55,56^ The thioesters were independently treated with ES and 3-MST, and the reaction mixtures were incubated with GSH followed by HPE-IAM treatment. Independently, a control experiment for 3-MST alone was carried out with GSH, and no significant persulfide generation was observed. We found that both mercapto-nabumetone derivatives were capable of generating GS-SH (Fig. 4C and D). AS was also used independently for the generation of persulfides, and a distinct HPEAM-S-SG signal was observed (Fig. S9†). In addition, bis-S-HPEAM, which is consistent with the formation of H_2_S, was also detected (Fig. 4E and S9, see the ESI†). In the absence of 3-MST, the mercapto-nabumetone adducts were unable to generate GS-SH and H_2_S adducts, suggesting the importance of 3-MST catalysis.

To better understand the reaction of 1 in the presence of ES, GSH, and 3-MST, HPLC analysis of this reaction mixture was carried out (Scheme S3, see the ESI†). Under these conditions, the transformation of 1 or 2 to nabumetone was observed, albeit at a lower efficiency compared to DTT (Fig. S10, see the ESI†). Previous reports suggest that GSH is not a good sulfur acceptor to regenerate the 3-MST enzyme in its active state.^35,46^ This lack of efficiency could contribute to diminished yields of nabumetone under these conditions. In the cellular milieu, enzymes such as thioredoxin reductase predominantly convert 3-MST persulfides to 3-MST and H_2_S.^46^ DTT is a mimic of thioredoxin reductase, and in our hands, mercapto-nabumetone derivatives are transformed to the NSAID in excellent yield.

To further analyse the production of H_2_S under reducing conditions, we used two independent assays. First, a methylene blue assay, where the aforementioned adduct of H_2_S has a distinct absorbance at 676 nm, was used (Scheme S6, see the ESI†).^29,46^ Under the standard reaction conditions, both 1 and 2 were found to generate H_2_S (Fig. 4F). A time course of H_2_S generation was recorded and curve fitting gave observed rate constants of 0.68 h^−1^ and 0.62 h^−1^, for 1 and 2, respectively (Fig. S11, see the ESI†).

These values are comparable with the observed rate constants of conversion of thiols, T1 or T2, to nabumetone (0.57 h^−1^ and 0.6 h^−1^). Hence, there appear to be no other byproducts during the transformation of these thiols into the parent NSAID.

To further validate H_2_S generation from α-mercapto-nabumetone derivatives 1 and 2, a lead acetate assay was carried out.^57^ A lead acetate solution-soaked paper was used to trap lead sulfide (black coloration), which is formed by the reaction of lead acetate and H_2_S (Scheme S7, see the ESI†). Both derivatives 1 and 2 gave an intense black coloration in this assay (Fig. 4G). Under similar conditions, no significant signal for H_2_S was observed with 4 or 5 (Fig. S12, see the ESI†).

### Cellular H_2_S generation and effect on cell viability

Having established that mercapto-nabumetone derivatives were able to generate persulfide, H_2_S, and the parent NSAID, we next tested if these derivatives were able to permeate cells to generate H_2_S. Mouse Embryonic Fibroblast (MEF) cells^58^ were co-treated with mercapto-nabumetone derivatives 1 or 2 as well as the H_2_S-sensitive fluorogenic NBD-fluorescein dye.^59^ The cells were imaged after 1 h of incubation using a green fluorescent protein (GFP) filter. A distinct fluorescent signal for 1 and 2 was observed, supporting the ability of these derivatives to enhance intracellular H_2_S in MEF cells (Fig. 4H). Derivative 2 has somewhat lower fluorescence intensity when compared with 1. This may be due to differences in permeability but could also be due to differences in the reactivity of the substrate with 3-MST *in cellulo*. The oxygen derivative 4 did not show any detectable fluorescence signal in this experiment (Fig. S13, see the ESI†).

Making modifications to a clinical drug can result in adverse effects on pharmacological properties. To understand the impact of an additional sulfhydryl group on cell viability in MEF cells, 1 and 2 were independently tested and compared with nabumetone. We found that much like nabumetone, neither derivative had any adverse effects on cell viability (Fig. S14, see the ESI†).

### Applicability of the strategy to other mercapto-NSAIDs

**Scheme 2:**
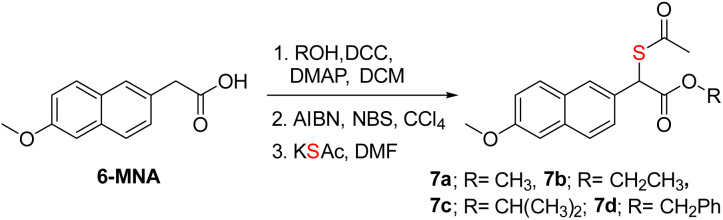
Synthesis of mercapto-6-MNA with different esters. Overall yields were 79–85%.

Previous strategies to produce NSAID and H_2_S have relied on making large-scale modifications to the NSAID. For example, the clinical agent ATB-346 has a thiobenzamide ring attached to the NSAID, which adds a number of carbons to the parent drug itself.^19,60^ The most atom-economical approach is to make the thioacid of the NSAID; such thioacids are hydrolyzed to generate H_2_S.^20^ The acidity of thioacids is significantly higher than that of carboxylic acids, and this may alter permeability and these compounds are likely to generate H_2_S outside as well as inside cells. Our approach is also similarly minimalistic, and the major modification is to add a thioacetate group and, in principle, a sulfhydryl group to generate H_2_S along with the NSAID. Since nabumetone is consumed orally, we compared the NSAID, T1 and T2 using SwissADME software (see the ESI†). We found that the physicochemical properties of the thiols are similar to those of nabumetone (Tables S2 and S3 and Fig. S15, see the ESI† for detailed physicochemical properties). Several NSAID derivatives have a free carboxylic acid,^13–15^ and we considered mercapto-6-MNA.^61^ 6-MNA is a product of metabolism of nabumetone and has excellent anti-inflammatory activity. We next prepared a series of 6-MNA ester derivatives with a thioacetate at the α-position (Schemes 2 and S8, see the ESI†).^62,63^ The carboxylic acid esters are expected to undergo hydrolysis to produce 6-MNA.

**Fig. 5:**
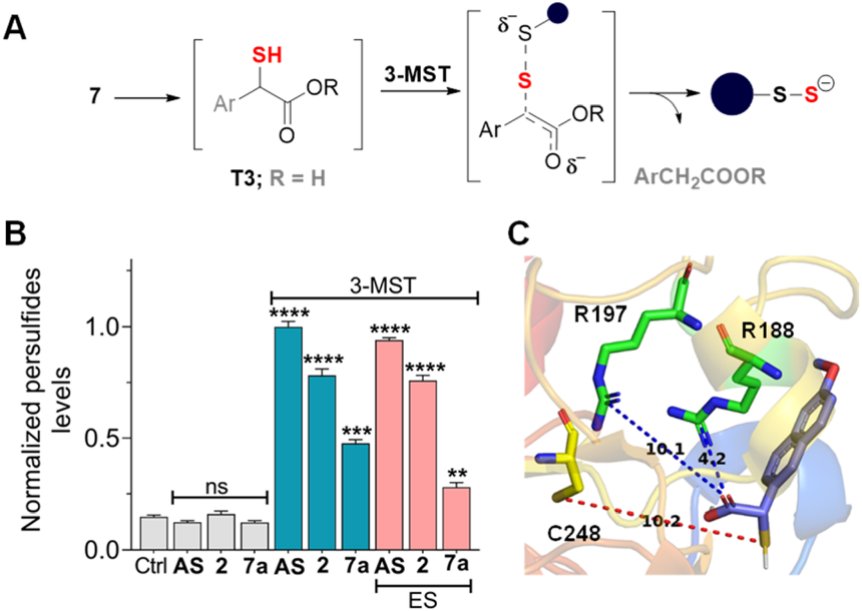
(A) Compounds 7a–7d are expected to produce the corresponding thiol (Ar = 6-methoxynaphthyl and R = various groups), which reacts with 3-MST to generate persulfide. When ES is added, the thiol T3 (Ar = 6-methoxynaphthyl) is the likely intermediate. (B) Sulfane sulfur detection in the presence of 3-MST (15 μM) and SSP-2 (50 μM). The assay was conducted both in the absence of ES and in the presence of ES. Persulfide generation is significantly higher in the absence of ES. Results are presented as mean ± SD (*n* = 3). Student's two-tailed unpaired parametric *t*-test was used to determine significance: ns = nonsignificant, ****p < 0.0001, ***p < 0.001, and **p < 0.01 with respect to 3-MST only. Ctrl represents 3-MST only. (C) Lowest energy conformations found through molecular docking studies with human 3-MST and T3. Thiols T1, T2, and T3 show comparable docking energy, *i.e.*, −5.4 kcal mol^−1^, −5.5 kcal mol^−1^, and −5.3 kcal mol^−1^, respectively. The distance between the sulfur of T3 and the sulfur of active residue C248 is 10.2 Å.

Compounds in series 7 should be cleaved by ES to produce T3, which, in the presence of 3-MST, should generate persulfide (Fig. 5A). First, 7a was reacted with ES and 3-MST, and the formation of persulfides was analysed by SSP-2 assay. This assay when conducted in the absence of ES, and a significant increase in persulfides for 7a was observed (Fig. 5B). A comparable result was observed with lead acetate as well (Fig. S16, see the ESI†). Although 7a produces persulfides and H_2_S, the yield was significantly lower than that of 2. Similar results were recorded for 7b–7d with both SSP-2 and lead acetate assays (Fig. S17 and S18, see the ESI†). These data suggest that the methyl ester was a better persulfide generator when compared with carboxylic acids and other ester groups. To further understand the ester group effect on binding with 3-MST, molecular docking was carried out with thiols of 7a–7d (Table S1, see the ESI†). Molecular docking analysis supports favorable binding of thiol T3 (in the carboxylic acid form) with the active site of 3-MST, similar to 2 (Fig. 5C and Table S1, see the ESI†). Hence, the diminished rate might be due to reduced catalysis.

The key step of C–S bond cleavage depends on the formation of an enol(ate), and in the case of 1 or 2, the enolate of a ketone is produced, while in the case of 6-MNA derivatives, an enolate of an ester is the likely intermediate (Fig. 5A). The keto–enol equilibria of ketones and esters suggest that their equilibrium constants (*K*) are in favor of the keto form but are quite different. The values of *K* for ketones range from 10^−7^ to 10^−8^. Although the position of the keto–enol equilibrium for nabumetone has not been experimentally determined, the closest structure, acetone, has a *K* of 10^−8^.^64^ The *K* for acetophenone, the product of the 3-MST catalysed reaction of phenacylthiol, is 10^−7^, which supports a relatively stable enol.^38^ Previously, we showed that the presence of an electron withdrawing group on the aromatic ring of phenacylthiol induced moderate acceleration of H_2_S generation through 3-MST catalysis.^38^ This is presumably due to the additional stability of the intermediary enolate that is formed during C–S bond cleavage (Fig. 5A).

The keto–enol *K* for an ester is estimated to be 10^−19^.^64–67^ These values are reflective of a less stable enol in the case of an ester, and hence, the transition state leading to the formation of the ester enolate (Fig. 5A) is likely to have a higher barrier. Yet, 3-MST is able to catalyse, in some cases, the transformation of a mercapto-ester to a persulfide. This result underscores the versatility of this enzyme and provides insights into the binding and catalysis by 3-MST (Table S1, see the ESI†). However, when the ester is hydrolysed to the carboxylate of T3, the reaction does not proceed well. 3-MP is also expected to be in the carboxylate form, but 3-MST catalysis proceeds efficiently. We infer that, in the case of T3, the anionic form (carboxylate) reduces the stability of the enolate, leading to reduced catalysis rates. Hence, enolate stability or lack therefor drives the reaction outcome. Further studies on the structure–enzyme activity relationship are necessary to better understand these aspects of 3-MST catalysis. Nevertheless, this strategy can be applicable to other NSAIDs that have a ketone functional group^68,69^ and perhaps an amide.^70,71^ Lastly, the mercapto-NSAID can be functionalized with suitable protective groups that can help with site-specific activation by enzymes or ROS to facilitate release of the NSAID as well as H_2_S.

## Conclusions

A new approach to modifying an NSAID to generate hydrogen sulfide has been developed. The introduction of a sulfhydryl group at the α-position of a ketone or an ester was found to be a viable strategy to generate H_2_S along with the parent carbonyl compound. The yield of the NSAID in the case of ketone was excellent (>90%), and the hybrids produced key cellular antioxidant response molecules, GS-SH and H_2_S. Since persulfides have superior antioxidant properties, these compounds are expected to have translational value. The α-mercapto-ester produced persulfide and H_2_S under these conditions but was less efficient than the ketone. Hence, enolate stability is a major contributor to sulfur transfer by 3-MST and will help with future design of artificial substrates for this enzyme. Taken together, we provide evidence for an atom economical approach to co-generating H_2_S and an NSAID, and further studies are underway to evaluate the therapeutic potential of these compounds.

## Data availability

The data supporting this article have been included as part of the ESI.†

## Author contributions

All experiments and computational studies were carried out by SMG and PSM, under the supervision of HC. The manuscript was written with inputs from all authors.

## Conflicts of interest

There are no conflicts to declare.

## Supplementary Material

SC-OLF-D4SC08525F-s001
