# Supplementary material for: Mercapto-NSAIDs generate a non-steroidal anti-inflammatory drug (NSAID) and hydrogen sulfide
Source: Chem Sci. 2025 Feb 5;16(11):4695–702. doi: 10.1039/d4sc08525f (PMC11826334; doi:10.1039/d4sc08525f)

Supplementary Information (SI) for Chemical Science.
This journal is © The Royal Society of Chemistry 2025

Electronic Supplementary Material (ESI) for Chemical Science

This journal is © The Royal Society of Chemistry 2024

Electronic Supplementary Information (ESI)

for

Mercapto-NSAIDs Generate a Non-Steroidal Anti-Inflammatory Drug (NSAID) and Hydrogen Sulfide

Simran M. Gupta,[a] Pratiksha S. Mohite, [a] and Harinath Chakrapani *[a]

[a]Department of Chemistry, Indian Institute of Science Education and Research Pune,

Dr. Homi Bhabha Road, Pashan Pune 411008, Maharashtra, India.

E-mail: harinath@iiserpune.ac.in

Table of contents

1	General methods	S3
2	Synthesis and characterization	S3
3	Experimental protocols	S9
4	Supplementary Schemes	S16
5	Supplementary Table	S18
6	Supplementary Figures	S21
7	References	S29
8	NMR spectra	S30

1. General methods

Unless otherwise specified, all reagents, starting materials, and dry solvents were purchased from commercial suppliers and used as received without further purification. All reactions were conducted under a nitrogen atmosphere. Thin-layer Chromatography (TLC) analyses were performed using pre-coated TLC sheets from MERCK® SIL G/UV254. Visualization was accomplished with UV light (λmax = 254 nm). Column chromatography was performed on Rankem silica gel (60-120 or 100-200 mesh). ^{1}H and ^{13}C NMR spectra were recorded either on BRUKER 400 MHz or JEOL 400 MHz (or 100 MHz for ^{13}C) spectrometers using tetramethylsilane (TMS, $\delta_H = 0.00$, $\delta_C = 0.00$) as an internal standard or residual solvent [$CHCl_3$ δ_H 7.26 ppm, δ_C 77.2 ppm] signals as reference. Chemical shifts (δ) are reported in parts per million (ppm) and coupling constants (*J*) in Hz. The following notations are used to indicate the multiplicity of the signals: s (singlet), d (doublet), t (triplet), dd (doublet of doublet), q (quartet), sep (septet), m (multiplet). High-resolution mass spectra (HRMS) were obtained from HRMS-ESI-Q-Time of Flight LC/MS. Infrared (IR) spectra were recorded using BRUKER-ALPHA FT-IR spectrometer. High-performance liquid chromatography (HPLC) was performed on an Agilent Technologies 1260 infinity with the Poroshell EC-C18 reverse phase column with 120 Å particle size and 2.7 µM pore size (150 × 3.0 m). Fluorescence and other spectrophotometric measurements were performed using EnSight Multimode Plate Reader under the Perkin Elmer-IISER Pune, Centre of Excellence facility.

2. Synthesis and characterization

Compounds **AS** (artificial substrate, phenacyl thioacetate),[1] NBD-Fluorescein,[2] and SSP-2[3] were synthesized as per reported protocols, and their analytical data were consistent with literature values.

1-Bromo-4-(6-methoxynaphthalen-2-yl)butan-2-one (1-Bromo-Nabumetone, 6a): From

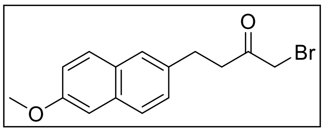

Nabumetone (**3**), 1-bromo-Nabumetone was synthesized using a reported protocol with some modifications.[4] **3** (300 mg, 1.3 mmol, 1 eq) was dissolved in $CHCl_3$/MeOH (1:1). To this solution, $CuBr_2$ (352 mg, 1.58 mmol, 1.2 eq)) added, and the mixture was heated to 40 °C for 8 h. Upon completion of the reaction (TLC analysis), the reaction mixture was quenched by adding water, and extracted with dichloromethane (DCM). The combined organic layer was again washed with brine, and the extract was dried over Na_2SO_4, filtered, and the filtrate was concentrated under reduced pressure to afford the crude product, which was purified by silica gel column chromatography (4-5% ethyl acetate/hexane) using 100-200 mesh silica as the

stationary phase. The desired product **6a** was obtained as a white solid (180 mg, 45%); ^{1}H NMR (400 MHz, $CDCl_3$): δ 7.67 (d, *J* = 8.6 Hz, 2H), 7.56 (s, 1H), 7.28 (dd, *J* = 8.4, 1.7 Hz, 1H), 7.15-7.10 (m, 2H), 3.91 (s, 3H), 3.85 (s, 2H), 3.06 (t, *J* = 3.8 Hz, 4H); ^{13}C NMR (100 MHz, $CDCl_3$): δ 201.5, 157.5, 135.6, 133.3, 129.2, 129.1, 127.5, 127.2, 126.5, 119.1, 105.8, 55.4, 41.6, 34.5, 30.0.

3-bromo-4-(6-methoxynaphthalen-2-yl)butan-2-one (3-Bromo-Nabumetone, 6b):[5] From

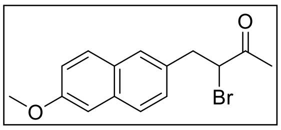

Nabumetone (**3**), 3-bromo-Nabumetone was synthesized using a reported protocol.[5] **3** (300 mg, 1.3 mmol, 1 eq.) was dissolved in THF. To this solution, phenyltrimethyl ammonium tribromide (PTT, 494 mg, 1.58 mmol, 1.2 eq) was added, and the mixture was stirred for 2 h at room temperature (RT). Upon completion of the reaction (TLC analysis), the reaction mixture was quenched by adding water, and extracted with DCM. The combined organic layer was again washed with brine, and the extract was dried over Na_2SO_4, filtered, and the filtrate was concentrated under reduced pressure to afford the crude product, which was purified by silica gel column chromatography (4-5% ethyl acetate/hexane) using 100-200 mesh silica as the stationary phase. The desired product **6b** was isolated as a white semi-solid (290 mg, 72%). ^{1}H NMR (400 MHz, $CDCl_3$): δ 7.70-7.67 (m, 2H), 7.58 (s, 1H), 7.28 (dd, *J* = 8.4, 1.8 Hz, 1H), 7.15-7.10 (m, 2H), 4.54 (t, *J* = 7.5 Hz, 1H), 3.91 (s, 3H), 3.57 (dd, *J* = 14.4, 7.6 Hz, 1H), 3.28 (dd, *J* = 14.4, 7.3 Hz, 1H), 2.31 (s, 3H). All other analytical data are consistent with previously reported data.

***S*-(4-(6-Methoxynaphthalen-2-yl)-2-oxobutyl) ethanethioate (1):** 1-bromo-Nabumetone

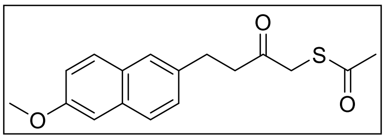

(**6a**) (180 mg, 0.59 mmol, 1 eq) was dissolved in anhydrous DMF. To this solution, potassium thioacetate (KSAc, 134 mg, 1.17 mmol, 2 eq) was added, and the reaction mixture was stirred for 2 h at RT. Upon completion of the reaction (TLC analysis), the reaction mixture was quenched by adding cold water, and extracted with ethyl acetate. The combined organic layer was again washed with brine, and the extract was dried over Na_2SO_4, filtered, and the filtrate was concentrated under reduced pressure to afford the crude product, which was purified by silica gel column chromatography (6-7% ethyl acetate/hexane) using 60-120 mesh silica as the stationary phase. The desired product **1** was isolated as a light brown solid (115 mg, 87%). FT-IR (υ_{max}, cm^{-1}): 1716, 1689; ^{1}H NMR (400 MHz, $CDCl_3$): δ 7.67 (d, *J* = 8.4 Hz, 2H), 7.55 (s, 1H), 7.27 (dd, *J* = 8.3, 1.7 Hz, 1H), 7.14-7.10 (m, 2H), 3.91 (s, 3H), 3.72 (s, 2H), 3.05-2.93 (m, 4H), 2.37 (s, 3H); ^{13}C NMR (100 MHz, $CDCl_3$): δ 203.4, 194.5, 157.5, 135.8, 133.3, 129.2, 129.1, 127.6, 127.2, 126.5, 119.0, 105.8, 55.4, 43.4, 39.3, 30.3, 29.8; HRMS for

$C_{17}H_{18}O_3S$ [M+Na]$^+$ Calculated: 325.0877, Found: 325.0874.

***S*-(1-(6-Methoxynaphthalen-2-yl)-3-oxobutan-2-yl) ethanethioate (2):** 3-Bromo-Nabumetone (**6b**) (290 mg, 0.94 mmol, 1 eq.) was dissolved in anhydrous DMF. To this solution, KSAc (215 mg, 1.9 mmol, 2 eq.) was added, and the reaction mixture was stirred for 2 h at RT. Upon completion of the reaction (TLC analysis), the reaction mixture was quenched by adding cold water, and extracted with ethyl acetate. The combined organic layer was washed with brine, and the extract was dried over Na_2SO_4, filtered, and the filtrate was concentrated under reduced pressure to afford the crude product. The crude was purified by silica gel column chromatography (6-7% ethyl acetate/hexane) using 60-120 mesh silica as the stationary phase. The desired product **2** was isolated as a light brown solid (190 mg, 67%). FT-IR (υ_{max}, cm^{-1}): 1713, 1695; ^{1}H NMR (400 MHz, $CDCl_3$): δ 7.68 (dd, *J* = 8.6, 4.7 Hz, 2H), 7.56 (s, 1H), 7.30 (dd, *J* = 8.4, 1.7 Hz, 1H), 7.15-7.10 (m, 2H), 4.60 (t, *J* = 7.6 Hz, 1H), 3.91 (s, 3H), 3.36 (dd, *J* = 14.0, 8.2 Hz, 1H) , 3.0 (dd, *J* = 14.0, 7.2 Hz, 1H), 2.32 (s, 3H), 2.13 (s, 3H); ^{13}C NMR (100 MHz, $CDCl_3$): δ 205.1, 194.3, 157.7, 133.6, 132.8, 129.0, 127.9, 127.8, 127.2, 119.1, 105.7, 55.4, 53.2, 36.5, 30.4, 29.3; HRMS for $C_{17}H_{18}O_3S$ [M+Na]$^+$ Calculated: 325.0877, Found: 325.0874.

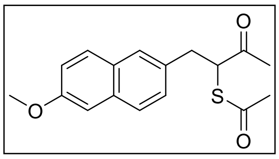

4-(6-Methoxynaphthalen-2-yl)-2-oxobutyl acetate (4): 1-Bromo-Nabumetone **(6a)** (100 mg, 0.32 mmol, 1 eq) was dissolved in anhydrous DMF. To this solution, sodium acetate (NaOAc, 53 mg, 65 mmol, 2 eq) was added, and the reaction mixture was stirred for 6 h at RT. Upon completion of the reaction (TLC analysis), the reaction mixture was quenched by adding cold water and extracted with ethyl acetate. The combined organic layer was again washed with brine, and the extract was dried over Na_2SO_4, filtered, and the filtrate was concentrated under reduced pressure to afford the crude product, which was purified by silica gel column chromatography (7-8% ethyl acetate/hexane) using 60-120 mesh silica as the stationary phase. The desired product **4** was isolated as a white solid (63 mg, 68%). FT-IR (υ_{max}, cm^{-1}): 1746, 1729; ^{1}H NMR (400 MHz, $CDCl_3$): δ 7.67 (d, *J* = 8.5 Hz, 2H), 7.55 (s, 1H), 7.28 (d, *J* = 1.6 Hz, 1H), 7.15-7.10 (m, 2H), 4.62 (s, 2H), 3.91 (s, 3H), 3.06 (t, *J* = 7.4 Hz, 2H), 2.81 (t, *J* = 7.4, 2H), 2.16 (s, 3H); ^{13}C NMR (100 MHz, $CDCl_3$): δ 203.3, 170.4, 157.6, 135.7, 133.4, 129.2, 129.1, 127.6, 127.3, 126.6, 119.1, 105.8, 68.3, 55.5, 40.7, 29.3, 20.7; HRMS for $C_{17}H_{18}O_4$ [M+H]$^+$ Calculated: 309.1097, Found: 309.1100.

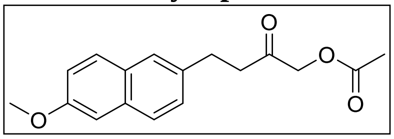

1-(6-Methoxynaphthalen-2-yl)-3-oxobutane-2-yl acetate (5): 3-Bromo-Nabumetone **(6b)**

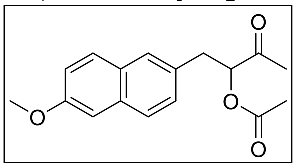

(100 mg, 0.33 mmol, 1 eq.) was dissolved in anhydrous DMF. To this solution, NaOAc (53 mg, 65 mmol, 2 eq) was added, and the reaction mixture was stirred for 6 h at RT. Upon completion of the reaction (TLC analysis), the reaction mixture was quenched by adding cold water, and extracted with ethyl acetate. The combined organic layer was again washed with brine, and the extract was dried over Na_2SO_4, filtered, and the filtrate was concentrated under reduced pressure to afford the crude product, which was purified by silica gel column chromatography (7-8% ethyl acetate/hexane) using 60-120 mesh silica as the stationary phase. The desired product **5** was isolated as a white solid (61 mg, 62%). FT-IR (υ_{max}, cm^{-1}): 1740, 1729; ^{1}H NMR (400 MHz, $CDCl_3$): 7.70-7.67 (m, 2H), 7.58 (s, 1H), 7.31 (dd, *J* = 8.4, 1.7 Hz, 1H), 7.16-7.11 (m, 2H), 5.27 (dd, *J* = 8.1, 5.0 Hz, 1H), 3.92 (s, 3H), 3.26-3.10 (m, 2H), 2.08 (s, 6H); ^{13}C NMR (100 MHz, $CDCl_3$): δ 205.6, 170.6, 157.8, 133.7, 131.1, 129.2, 129.0, 128.0, 127.3, 119.2, 105.8, 79.3, 55.5, 36.9, 29.8, 27.2, 20.8; HRMS for $C_{17}H_{18}O_4$ $[M+H]^+$ Calculated: 309.1097, Found: 309.1100.

General procedure for the synthesis of 9a-9d: From 6-Methoxy-2-naphthylacetic acid (6-MNA), **9a-9d** was synthesized using a reported protocol with some modifications.[6,7] Crude was taken to the next step without further purification.

General procedure for the synthesis of 8a-8d: From **9a-9d**, **8a-8d** were synthesized using a reported protocol with some modifications.[8] Azobisisobutyronitrile (AIBN, 0.1 eq) and *N*-bromosuccinimide (NBS, 1.2 eq) were added in a solution of **9a-9d** (1 eq) in CCl_4 (3 mL) at 70 ^{o}C. After stirring for 12 h, complete consumption of the starting material was monitored by TLC, the reaction was quenched by adding water, and the resulting solution was extracted with DCM. The combined organic phase was washed with brine, dried over Na_2SO_4, filtered, and the filtrate was evaporated under reduced pressure. This crude was further purified by column chromatography using silica gel 100-200 to obtain compounds **8a-8b** and **8c-8d** were used in the next step without further purification.

Methyl 2-bromo-2-(6-methoxynaphthalen-2-yl)acetate (8a): Following the general synthesis

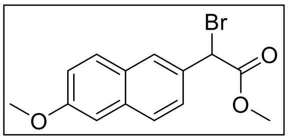

protocol mentioned above, **9a** was used as starting material (96 mg, 0.42 mmol) and dissolved in CCl_4 (3 mL). AIBN (6.85 mg, 0.04 mmol) and NBS (89.05 mg, 0.5 mmol) were sequentially added to this solution. After extraction, the crude product was purified by silica gel column chromatography using 3-4% ethyl acetate/hexane. The desired product **8a** was obtained as a white solid (90 mg, 70%).

^{1}H NMR (400 MHz, $CDCl_3$): δ 7.87 (d, J = 1.7 Hz, 1H), 7.73 (t, J = 8.3 Hz, 2H), 7.65 (dd, J = 8.6, 1.8 Hz, 1H), 7.18-7.12 (m, 2H), 5.52 (s, 1H), 3.92 (s, 3H), 3.80 (s, 3H); ^{13}C NMR (100 MHz, $CDCl_3$): δ 169.1, 158.8, 135.0, 130.8, 129.9, 128.5, 128.0, 127.9, 126.6, 119.7, 105.9, 55.6, 53.6, 47.4.

Ethyl 2-bromo-2-(6-methoxynaphthalen-2-yl)acetate (8b): Following the general synthesis

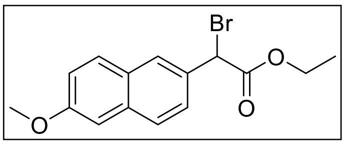

protocol mentioned above, **9b** was used as starting material (77 mg, 0.31 mmol) and dissolved in CCl_4 (3 mL). AIBN (5.18 mg, 0.03 mmol) and NBS (67.32 mg, 0.38 mmol) were sequentially added to this solution. After extraction, the crude product was purified by silica gel column chromatography using 3-4% ethyl acetate/hexane. The desired product **8b** was obtained as a white solid (50 mg, 49%). ^{1}H NMR (400 MHz, $CDCl_3$): δ 7.87 (d, J = 1.3 Hz, 1H), 7.73 (t, J = 8.1 Hz, 2H), 7.66 (dd, J = 8.7, 1.8 Hz, 1H), 7.18-7.12 (m, 2H), 5.50 (s, 1H), 4.28-4.15 (m, 2H), 3.92-3.91 (m, 3H), 1.31-1.25 (m, 3H); ^{13}C NMR (100 MHz, $CDCl_3$): δ 168.5, 158.7, 135.0, 130.9, 129.9, 128.5, 127.9, 127.8, 126.6, 119.6, 105.8, 62.7, 55.5, 47.7, 14.1.

Isopropyl 2-bromo-2-(6-methoxynaphthalen-2-yl)acetate (8c): Following the general

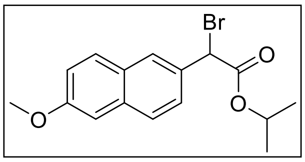

synthesis protocol mentioned above, **9c** was used as starting material (114 mg, 0.44.mmol) and dissolved in CCl_4 (3 mL). To this solution, AIBN (7.25 mg, 0.04 mmol) and NBS (94.26 mg, 0.53 mmol) were added to obtain compound **8c** (44 mg, 30%). Compound **8c** was taken for the next step without any further purification.

Benzyl 2-bromo-2-(6-methoxynaphthalen-2-yl)acetate (8d): Following the general synthesis

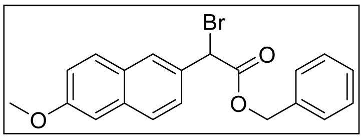

protocol mentioned above, **9d** was used as starting material (20 mg, 0.06mmol) and dissolved in CCl_4 (3 mL). To this solution, AIBN (1.05 mg, 0.006 mmol) and NBS (13.94 mg, 0.08 mmol) were added to obtain compound **8d** (15 mg, 60%). Compound **8d** was taken for the next step without any further purification.

General procedure for the synthesis of 7a-7d: From **8a-8d**, **7a-7d** were synthesized using a reported protocol.[1] KSAc (2 eq) was added in a solution of **8a-8d** (1 eq) in DMF at RT. After stirring for 1 h, the complete consumption of the starting material was monitored by TLC. The reaction mixture was quenched by adding cold water, and extracted with ethyl acetate. The combined organic phase was washed with brine, dried over Na_2SO_4, filtered, and the filtrate was evaporated under reduced pressure. This crude was further purified by column chromatography

using silica gel (60-120) to obtain compound **7a-7d.**

Methyl 2-(acetylthio)-2-(6-methoxynaphthalen-2-yl)acetate (7a): Following the general

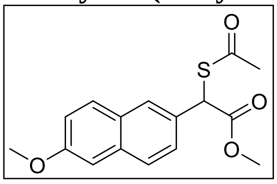

synthesis protocol mentioned above, **8a** was used as starting material (90 mg, 0.30 mmol) and dissolved in DMF (4 mL), followed by the addition of KSAc (66.49 mg, 0.60 mmol). After extraction, the crude was purified by silica gel column chromatography using 5-6% ethyl acetate/hexane. The desired product **7a** was obtained as a brown solid (72 mg, 81%). FT-IR (υ_{max}, cm^{-1}): 1739, 1694; ^{1}H NMR (400 MHz, $CDCl_3$): δ 7.78 (d, *J* = 1.5 Hz, 1H), 7.71 (d, *J* = 8.6 Hz, 2H), 7.43 (dd, *J* = 8.5, 1.8 Hz, 1H), 7.15 (dd, *J* = 8.9, 2.5 Hz, 1H), 7.10 (d, *J* = 2.4 Hz, 1H), 5.46 (s,1H), 3.91 (s, 3H), 3.75 (s, 3H), 2.36 (s, 3H); ^{13}C NMR (100 MHz, $CDCl_3$): δ 194.2, 170.8, 158.4, 134.5, 129.8, 129.7, 128.8, 127.8, 127.6, 126.4, 119.6, 105.8, 55.5, 53.3, 51.4, 30.2; HRMS for $C_{16}H_{16}O_4S$ $[M+Na]^+$ Calculated: 327.0666, Found: 327.0665.

Ethyl 2-(acetylthio)-2-(6-methoxynaphthalen-2-yl)acetate (7b): Following the general

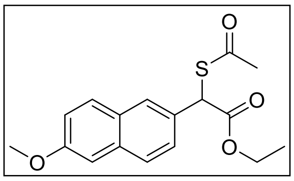

synthesis protocol mentioned above, **8b** was used as starting material (50 mg, 0.15 mmol) and dissolved in DMF (4 mL), followed by the addition of KSAc (35.34 mg, 0.30 mmol). After extraction, the crude was purified by silica gel column chromatography using 5-6% ethyl acetate/hexane. The desired product **7b** was obtained as a yellow solid (42 mg, 85%). FT-IR (υ_{max}, cm^{-1}): 1735, 1693; ^{1}H NMR (400 MHz, $CDCl_3$): δ 7.79 (d, *J* = 1,3 Hz, 1H), 7.71 (dd, *J* = 8.7, 1.8 Hz, 2H), 7.43 (dd, *J* = 8.4, 1.9 Hz, 1H), 7.15 (dd, *J* = 8.9, 2.5 Hz, 1H), 7.10 (d, *J* = 8.9 Hz, 1H), 5.44 (s, 1H), 4.27-4.14 (m, 2H), 3.91 (s, 3H), 2.36 (s, 3H), 1.24-1,22(m, 3H); ^{13}C NMR (100 MHz, $CDCl_3$): δ 194.2, 170.2, 158.3, 134.5, 130.1, 129.7, 127.8, 127.6, 126.5, 119.6, 105.8, 62.3, 55.5, 51.6, 30.2, 14.2; HRMS for $C_{17}H_{18}O_4S$ $[M+Na]^+$ Calculated: 341.0823, Found: 341.0825.

Isopropyl 2-(acetylthio)-2-(6-methoxynaphthalen-2-yl)acetate (7c): Following the general

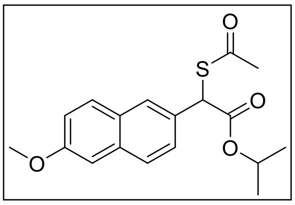

synthesis protocol mentioned above, **8c** was used as starting material (44 mg, 0.13 mmol) and dissolved in DMF (4 mL), followed by the addition of KSAc (29.8 mg, 0.26 mmol). After extraction, the crude was purified by silica gel column chromatography using 5-6% ethyl acetate/hexane. The desired product **7c** was obtained as a yellow solid (35.5 mg, 82%). FT-IR (υ_{max}, cm^{-1}): 1730, 1694; ^{1}H NMR (400 MHz, $CDCl_3$): δ 7.78 (d, *J* = 1.4 Hz, 1H), 7.71 (dd, *J* = 8.7, 1.8 Hz, 2H), 7.44 (dd, *J* = 8.4, 1.9 Hz, 1H), 7.15 (dd, *J* = 8.9, 2.5 Hz, 1H), 7.10 (d, *J* = 8.9 Hz, 1H), 5.40 (s, 1H), 5.05 (sep, *J* = 6.3 Hz, 1H), 3.91 (s, 3H), 2.35 (s, 3H), 1.28 (d, *J* = 6.2 Hz,

3H), 1.15 (d, J = 6.2 Hz, 3H); ^{13}C NMR (100 MHz, $CDCl_3$): δ 194.3, 169.6, 158.3, 134.4, 130.2, 129.7, 128.8, 127.7, 127.5, 126.4, 119.5, 105.8, 70.0, 55.5, 51.8, 30.2, 21.8, 21.6; HRMS for $C_{18}H_{20}O_4S$ $[M+Na]^+$ Calculated: 355.0979, Found: 355.0974.

Benzyl 2-(acetylthio)-2-(6-methoxynaphthalen-2-yl)acetate (7d): Following the general synthesis protocol mentioned above, **8d** was used as starting material (15 mg, 0.04 mmol) and dissolved in DMF (2 mL), followed by the addition of KSAc (8.89 mg, 0.08 mmol). After extraction, the crude was purified by silica gel column chromatography using 4-5% ethyl acetate/hexane. The desired product **7d** was obtained as a pale yellow solid (11 mg, 79%). FT-IR ($υ_{max}$, cm^{-1}): 1738, 1690; ^{1}H NMR (400 MHz, $CDCl_3$): δ 7.74 (s, 1H), 7.70-7.65 (m, 2H), 7.41 (dd, J = 8.5, 1.8 Hz, 1H), 7.30-7.27 (m, 5H), 7.14 (dd, J = 8.8, 2.5 Hz, 1H), 7.10 (d, J = 2.4 Hz, 1H), 5.50 (s, 1H), 5.19 (dd, J = 35.9, 12.4 Hz, 2H), 3.91 (s, 3H), 2.36 (s, 3H); ^{13}C NMR (100 MHz, $CDCl_3$): 194.2, 170.1, 158.4, 129.8, 129.7, 128.8, 128.7, 128.5, 128.2, 127.8, 127.7, 126.5, 119.5, 105.8, 67.9, 55.5, 51.6, 29.9; HRMS for $C_{22}H_{20}O_4S$ $[M+Na]^+$ Calculated: 403.0979, Found: 403.0983.

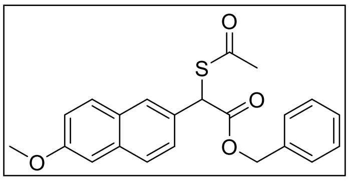

3. Experimental protocols

3.1. Docking methods for *h*3-MST

Autodock vina (V. 1.1.2) software was used to perform the docking of compounds (**T1**, **T2**, and **T3**) to the active site of ***h*3-MST** protein data bank (PDB) file: **4JGT**.[9] Molecules were converted to PDB from PyMOL and then used for docking in Chimera and AutoDock Vina (**Figure 2** and **Table S1**).[10] Docking was performed using an exhaustiveness parameter of 40 and keeping all other parameters to the vina default. The obtained file was saved as a PDBQT file to open in PyMOL for image analysis. The parameters defining the docking site in Chimera are:

Box Coordinates: Centre**:** (127, -35.318, 5.183), which is nearest (<1.6 Å) to the CG sidechain carbon of the ARG-188, ARG-197, CYS-248 residue of **4JGT**.

Box Dimensions (Å): 22 × 24 × 22.5

3.2. HPLC-based studies

General method: A fresh stock solution of **1** and **2** (10 mM) was prepared in DMSO, whereas the stocks of dithiothreitol (DTT, 100 mM), glutathione (GSH, 50 mM), and porcine liver esterase (ES, 100 U/mL; Sigma Aldrich, E3019) were prepared freshly in deionized (DI) water.

A stock of 285 μM of 3-MST was used. The reaction mixtures were prepared in 1x PBS (10 mM, pH 7.4) and placed in a shaker incubator maintained at 37 °C and 300 rpm. The final concentration of DMSO in the reaction mixture was at 1%. At pre-determined time points, aliquots were taken from the reaction mixture, and the reaction was quenched, half diluted with acetonitrile (ACN), centrifuged, and injected (25 μL) in HPLC attached with a UV detector (absorbance at 280 nm). The Poroshell EC-C18 reverse phase column was used with 120 Å particle size and 2.7 μm pore size (150 × 3.0 mm). The mobile phase was water: ACN with a gradient starting at 55:45 to 45:55 → 0 – 3 min, 45:55 to 30:70 → 3 - 5 min, 30:70 to 20:80 → 5 - 8 min, 20:80 to 30:70 → 8 - 10 min, 30:70 to 45:55 → 10 – 13 min, 44:55 to 55:45 → 13 - 18 min was used at a flow rate of 0.5 mL/min.

3.2.1. Stability studies under non-reducing conditions.

A 10 mM stock solution of **1** and **2** was prepared in DMSO. The reaction mixture contained 100 μM of **1** or **2** (10 μL, 10 mM) and the remaining volume was adjusted to 1000 μL in a 1.5 mL vial with 1x PBS pH 7.4 buffer (**Figure S1**) or 1x PBS pH 2.0 buffer (**Figure S5**) 1x PBS pH 6.8 buffer (**Figure S6**). The reactions were placed in a shaker incubator maintained at 37 ºC and 300 rpm. 200 μL aliquots were quenched with ACN, and 25 μL of the sample was analyzed by HPLC

3.2.2. Cleavage of 1 and 2 in the presence of ES under non-reducing conditions.

The reaction mixture was prepared using 100 µM of **1** or **2** (10 µL, 10 Mm), with 1 U/mL of ES (10 µL, 100 U/mL), and the volume was adjusted to 1000 µL using 1x PBS pH 7.4 buffer in a 1.5 mL vial. The mixture was then incubated at 37 ºC for 90 min. 200 μL aliquots were taken out from the reaction mixture, diluted with 200 μL ACN, and 25 μL of the sample was analyzed by HPLC (**Figure S2**).

3.2.3. Cleavage of 1 and 2 in the presence of ES under reducing conditions.

The reaction mixture was prepared using 100 µM of **1** or **2** (10 µL, 10 mM), with 1 U/mL of ES (10 µL, 100 U/mL), 10 mM of DTT (100 µL, 100 mM), and volume was adjusted to 1000 µL using 1x PBS pH 7.4 buffer in a 1.5 mL vial. The mixture was then incubated at 37 ºC for 90 min. 200 μL aliquots were taken out from the reaction mixture and diluted with the same volume of ACN, and 25 μL of the sample was analyzed by HPLC (**Figure S4**).

3.2.4 Turnover of T1 and T2 in the presence of DTT to produce Nabumetone (3).

The reaction mixture was prepared using 100 µM of **1** or **2** (30 µL, 10 mM), 1 U/mL of ES (30 µL, 100 U/mL), 10 mM of DTT (300 µL, 100 mM), and 1 µM of 3-MST (10.6 µL, 285

µM), and volume was adjusted to 3 mL using 1x PBS pH 7.4 buffer. The mixture was then incubated at 37 °C and 300 rpm. At pre-determined time points, 200 µL aliquots were taken from the reaction mixture quenched with 200 µL ACN, and 25 µL of the sample was analyzed by HPLC (**Scheme S3** and **Figure S7**).

3.2.5 Turnover of T1 and T2 in the presence of GSH to produce Nabumetone (3).

The reaction mixture was prepared using 100 µM of **1** or **2** **(**30 µL, 10 mM), 1 U/mL of ES (30 µL, 100 U/mL), 2 mM of GSH (120 µL, 50 mM), and 1 µM of 3-MST (10.6 µL, 285µM), and volume was adjusted to 3 mL using 1x PBS pH 7.4 buffer. The mixture was then incubated at 37 °C at 300 rpm. At pre-determined time points, 200 µL aliquots were taken from the reaction mixture, diluted with 200 µL ACN, and 25 µL of the sample was analyzed by HPLC (**Scheme S3** and **Figure S10**).

3.3. MALDI-TOF analysis

Trapping experiment with monobromobimane (mBBr).

Decomposition of 1 in the presence of ES to give T1, which forms an adduct with mBBr: Stock solutions of **1** and mBBr (10 mM) in DMSO, and ES (100 U/mL; Sigma Aldrich, E3019) in DI water were prepared. The reaction mixture was prepared by adding 100 µM of **1** (10 µL, 10 mM), 1 U/mL of ES (10 µL, 100 U/mL stock), 100 µM of mBBr (10 µL, 10 mM), and the volume was adjusted to 1000 µL using 1x PBS pH 7.4 buffer in a 1.5 mL vial and then incubated at 37 °C for 30 min. The 200 µL reaction mixture was then extracted with an equal volume of ethyl acetate. The resultant organic layer was submitted to MALDI-TOF for the analysis of adducts. The expected mass for **T1-S-bimane** adduct (expected m/z =450.5530, $[M+H]^+$ = 451.5608, $[M+Na]^+$ = 473.5427, $[M+K]^+$ = 489.6313) (**Scheme S2, Figure S3**).

3.4. Persulfide/polysulfide detection using SSP-2.[11]

Stock solutions of **AS**, **1**, **2**, **4**, **5**, **7a-7d** (10 mM), and **SSP-2** (5 mM) were prepared in DMSO, ES (100 U/mL) in DI water, and DTT (100 mM) were prepared in 1x PBS pH 7.4 buffer. Individual reaction samples were prepared for **AS, 1, 2, 4,** or **5** by adding 15 µM of 3-MST (39.6 µL, 265 µM), 100 µM of compound (7 µL, 10 mM) and 1 U/mL of ES (7 µL, 100 U/mL**)**. The volume was adjusted to 700 µL using 1x PBS buffer (10 mM, pH 7.4). The control reaction mixtures were prepared by adding 15 µM of 3-MST (39.6 µL, 265 µM) and 7 µL of DMSO, and the volume was adjusted to 700 µL using 1x PBS pH 7.4 buffer. Compound controls were prepared by adding 100 µM of compound (7 µL, 10 mM) in 1 U/mL of ES (7 µL, 100 U/mL), and the volume was adjusted to 700 µL using 1x PBS pH 7.4 buffer. The

reaction mixtures were then placed on a shaker incubator maintained at 37 °C and 300 rpm for 2 h.

A similar reaction was set up for DTT control. Individual reaction samples were prepared for **AS**, **1**, and **2**. The reaction mixtures were prepared by adding 100 µM of compound (7 µL,10 mM) with 1 U/mL of ES (7 µL,100 U/mL), 15 µM of 3-MST (39.6 µL, 265 µM), and volume was adjusted to 700 µL using 1x PBS pH 7.4 buffer. The reaction mixture was then incubated at 37 °C and 300 rpm for 2 h. Finally, the reaction mixture was treated with 10 mM DTT (70 µL, 100 mM stock) after 30 min and further incubated at 37 °C for 2 h.

SSP-2 assay was also performed with **7a-7d** derivatives where individual reaction samples were prepared by adding 15 µM of 3-MST (28µL, 392 µM), 100 µM of compounds (7 µL, 10 mM), in the presence or absence of 1 U/mL of ES (7 µL, 100 U/mL). Next, the volume was adjusted to 700 µL using 1x PBS pH 7.4 buffer. The control reaction mixtures were prepared by adding 15 µM of 3-MST in 7 µL of DMSO, and the volume was adjusted to 700 µL using 1x PBS pH 7.4 buffer. Compound controls were prepared by adding 100 µM of compound (7 µL, 10 mM) in 1 U/mL of ES (7 µL, 100 U/mL), and the volume was adjusted to 700 µL using 1x PBS pH 7.4 buffer The reactions were then placed on a shaker incubator maintained at 37 °C and 300 rpm for 2 h.

The above treatment groups were finally incubated with 50 µM of SSP-2 (7 µL, 5 mM) at 37 °C for 15 min in the dark. 100 µL aliquot of each sample was transferred to a 96-well plate, and the fluorescence was recorded (λ_{ex} = 482 nm, λ_{em} = 518 nm) using a microplate reader (Thermo Scientific VarioskanFlash) (**Scheme S4**, **Figures S8** and **S17**).

3.5. Persulfide and hydrogen sulfide measurement using LC-MS.[12]

Trapping Experiments with β-hydroxyphenyl-ethyl-Iodoacetamide (HPE-IAM).

Stock solutions of **1** or **2** (10 mM) and HPE-IAM (10 mM) were prepared in DMSO. GSH (50 mM) and ES (100 U/mL) were prepared in DI water. The reaction mixture was prepared by adding 25 µM of 3-MST (13 µL, 392 µM stock), 100 µM of compounds (2 µL, 10 mM), and 1 U/mL of ES (1 µL, 100 U/mL). Next, the volume was adjusted to 200 µL using 1x PBS pH 7.4 buffer. An enzyme control was also prepared by adding 25 µM of 3-MST (13 µL, 392 µM stock), and the volume was adjusted to 200 µL using 1x PBS pH 7.4 buffer. The reaction mixtures were incubated at 37 ºC and 300 rpm for 1 h. 400 µM of GSH (1.6 µL, 50 mM stock) was then added and incubated at 37 °C for 30 min. 1 mM of HPE-IAM (20 µL, 10 mM stock) was added to the reaction mixtures and incubated further at 37 ºC and 300 rpm for 30 min. Finally, the reaction mixtures were diluted using 200 µL ACN, the samples were centrifuged at 10000×g for 10 min, and the supernatants were collected and assessed thereafter by LC/MS. 10 µL of the reaction

extract was injected into an Agilent 6545 LC-QTOF (quadrupole-time-of-flight) LC-MS/MS for semi-quantitative analysis using high-resolution auto MS-MS methods and chromatography techniques. A Gemini 5U C-18 column (Phenomenex) coupled with a Gemini guard column (Phenomenex, 4x3 mm, Phenomenex security cartridge) was used for LC separation. The solvents were - A: H_2O + 0.1% Formic Acid and B: ACN. Methods were 15 min long, starting with 0.1 mL/min 100% buffer A for 1.5 min, 0.5 mL/min linear gradient to 100% buffer B over 4.5 min, 0.5 mL/min 100% buffer B for 5.5 min, and equilibration with 0.5 mL/min 100% buffer A for 3.5 min. The MS parameters (positive mode ESI-MS) used were as follows: drying and sheath gas temperature = 350 °C; drying and sheath gas flow rate = 11L/min; fragment or voltage = 100 V; capillary voltage = 4 kV; nebulizer (ion source gas) pressure = 45 Ψ and nozzle voltage = 1 kV. For analysis, a custom library was employed in the form of a Personal Compound Database Library (PCDL), and the peaks were validated based on relative retention times and ppm errors.

The MRM-HR mass spectrometry parameters for measuring glutathione adduct (HPE-AM-SG) adduct ion mass (Q1, $M + H^+$) = 485.0777, glutathione persulfide (GSSH) adduct (HPE-AM-SSG) adduct ion mass (Q1, $M + H^+$) = 517.1427, and hydrogen sulfide (H_2S) adduct (HPE-AM-S-HPE-AM) adduct ion mass (Q1, $M + H^+$) = 389.1530, declustering potential =130 V, entrance potential = 10 V, collision energy = 33 V, and collision exit potential = 10 V. Area under the curve were then plotted for all the species (**Scheme S5**, **Figures S9A** and **S9B**).

3.6. Methylene blue assay for the detection of H_2S.[13,14]

The methylene blue assays were conducted as previously reported with some modifications. Briefly, 285 µM stock of 3-MST was used for the methylene blue assay. Stock solutions of all compounds **1** or **2** (10 mM) were prepared in DMSO. ES (100 U/mL) and $Zn(OAc)_2.2H_2O$ (40 mM) were prepared in DI water. DTT (100 mM) was prepared in 1x PBS pH 7.4 buffer. Stock solution of $FeCl_3$ (30 mM) was prepared in 1.2 M HCl, and *N*,*N*–dimethyl-*p*-phenylenediamine sulfate (DMPPDA, 20 mM) was prepared in 7.2 M HCl (methylene blue reagents).

Individual reaction samples were prepared for **1** or **2**. 100 µM of compound (15 µL, 10 mM) was added to 1 U/mL of ES (15 µL, 100 U/mL), 400 µM of $Zn(OAc)_2.2H_2O$ (15 µL, 40 mM), 1 µM of 3-MST (5.2 µL, 285 µM), and 10 mM of DTT (150 µL, 100 mM). The final volume was adjusted to 1.5 mL using 1x PBS pH 7.4 buffer and incubated at 37 °C and 300 rpm. At predetermined time intervals, an equal volume of 200 µL $FeCl_3$, DMPPDA, and aliquots from the above reaction samples were mixed and incubated at 37 °C for 30 min in the dark to allow the formation of the methylene blue dye. An aliquot of 150 µL was transferred to a 96-well plate, and the absorbance values were recorded at 676 nm using a microplate reader (Thermo

Scientific VarioskanFlash) (**Scheme S6** and **Figure S11**).

3.7. Lead Acetate assay for the detection of H_2S.[1]

The lead acetate assay was conducted as a previously reported method. Firstly, lead acetate socked paper was prepared using Whatman filter papers, which were soaked with 5% (w/v) lead (II) acetate solution and dried. Stocks of all compounds **AS**, **1**, **2**, **4**, **5**, and **7a-7d** (10 mM) were prepared in DMSO. ES (100 U/mL) in DI water and DTT (100 mM) was prepared in 1x PBS pH 7.4 buffer.

Lead acetate assay was performed in a 96-well plate (with lid). Individual reaction samples were prepared for **AS**, **1**, **2**, **4**, and **5**. 100 µM of compounds (10 µL, 10 mM) was subsequently added to 1 U/mL of ES (10 µL, 100 U/mL), 1 µM of 3-MST (2.7 µL, 285 µM) and 10 mM DTT (100 µL, 100 mM). The final volume was adjusted to 1000 µL using 1x PBS pH 7.4 buffer. A similar protocol was followed for the substrate controls using100 µM compounds (10 µL, 10 mM) was subsequently added to 1 U/mL of ES (10 µL, 100 U/mL), and 10 mM of DTT (100 µL, 100 mM). The final volume was adjusted to 1000 µL using 1x PBS buffer pH 7.4 buffer.

A separate experiment was conducted to test the enzyme controls. DMSO (1%), 3-MST (1 µM), and DTT (10 mM) were sequentially added. The final volume was adjusted to 1 mL using 1x PBS pH 7.4 buffer. All the reaction mixtures were covered with 5% lead acetate-soaked paper, sealed, and incubated at 37 °C for 2 h (**Scheme S7**).

The lead acetate assay was also conducted for **7a-7d** with the same procedure as mentioned above. 100 µM compounds of (10 µL, 10 mM) was subsequently added to with or without 1 U/mL ES (10 µL, 100 U/mL), 1 µM of 3-MST (2.6 µL, 392 µM) and 10 mM of DTT (100 µL, 100 mM). The final volume was adjusted to 1000 µL using 1x PBS pH 7.4 buffer, covered with 5% lead acetate-soaked paper, sealed, and incubated at 37 °C for 2.5 h. (**Scheme S7**).

A similar protocol was followed for the substrate controls. 100 µM of compounds (10 µL, 10 mM) was subsequently added to 1 U/mL of ES (10 µL, 100 U/mL), and 10 mM of DTT (100 µL, 100 mM). The final volume was adjusted to 1000 µL using 1x PBS pH 7.4 buffer, covered with 5% lead acetate-soaked paper, sealed, and incubated at 37 °C for 2.5 h. A separate experiment was conducted to test the enzyme controls. DMSO (1%), 3-MST (1 µM), and DTT (10 mM) were sequentially added. The final volume was adjusted to 1 mL using 1x PBS pH 7.4 buffer, covered with 5% lead acetate-soaked paper, sealed, and incubated at 37 °C for 2.5 h.

Lead acetate paper was carefully taken, and an image was captured using Syngene G-Box Chemi-XRQ. Image analysis using was carried out (**Figures S12**, **S16**, and **S18**).

3.8. Evaluation of physiochemical characteristics.[15]

Using SwissADME software (http://www.swissadme.ch/), **T1** and **T2** were evaluated along with Nabumetone (**Table S2** and **Figure S17**). The number of hydrogen bond donors (nHBD), topological polar surface area (TPSA), number of heavy atoms (nHA), number of aromatic heavy atoms (nAHA), molar refractivity (MR), and the number of hydrogen bond acceptors (nHBAs) for **T1** and **T2** were calculated using SwissADME software (**Table S2**). Lipophilicity properties of mercapto-nabumetone derivatives **T1** and **T2** were also calculated **(Table S3)**. Both derivatives **T1** and **T2** feature 5 and 4 rotatable bonds, respectively, which were nearly similar to Nabumetone. Consistent with the introduction of an electronegative sulfur atom, the TPSA for **T1** and **T2** are higher when compared with Nabumetone. Overall, the mercapto-NSAID derivatives and Nabumetone had similar physicochemical properties (**Figure S15**).

3.9. H_2S imaging in cells using NBD-fluorescein[2]

Mouse Embryonic Fibroblasts (MEF) cells were seeded in a 6-well plate with 10^5 cells/well in Dulbecco's modified eagle media (DMEM; HiMedia AL007A) supplemented with 10% Fetal Bovine Serum (FBS; Gibco) and 1% antibiotic solution and incubated in an atmosphere of 5% CO_2 at 37 °C for 12 h. After incubation, the old media was removed, and the cells were washed with 1 mL sterile 1x PBS pH 7.4 buffer. This was followed by the addition of 1 mL of fresh DMEM media having co-treated with compounds (100 µM) and NBD-fluorescein dye (10 µM) in cells and cells were incubated at 37 °C for 1 h. After 1 h, cells were washed twice with 1x PBS pH 7.4 buffer and then imaged under EVOS fluorescence microscope using 20x GFP (green fluorescence protein) filter (**Figure S13**).

3.10. Cell viability assay with the MEF cells

MEF cells were seeded in a 96-well plate at a concentration of 1×10^4 cells/well overnight in DMEM media supplemented with 10% FBS and 1% antibiotic solution in an atmosphere of 5% CO_2 at 37 °C. Following this, the cells were exposed to varying concentrations of compounds. The stock solutions of compounds **1, 2,** and **3** (10 mM) were prepared in DMSO**,** and the concentration of DMSO did not exceed 1% for all treatments. The cells were incubated for 16 h at 37 °C. Following this, the old media from the cells was removed, and a 0.5 mg/mL stock solution of 3-(4,5-dimethylthiazol-2-yl)-2,5-diphenyl tetrazolium bromide (MTT) was prepared in 7 mL DMEM media; 100 µL of the MTT stock was added to each well and incubated for 4 h at 37 °C. Next, the media was carefully removed, and 100 µL of DMSO was added to each well and allowed to mix correctly. Spectrophotometric analysis of each well at 570 nm was carried out using a microplate reader (Thermo Scientific Varioskan) to estimate cell viability (**Figure**

S14).

4. Supplementary Schemes

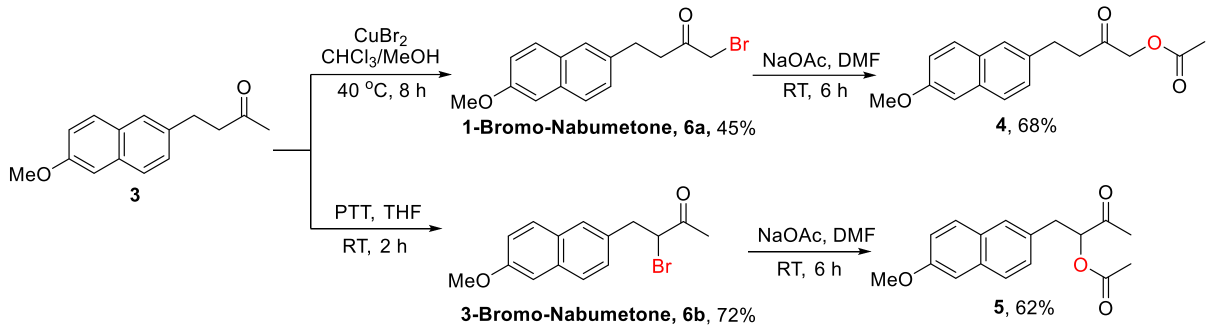

Scheme S1. Synthesis of negative controls **4** and **5**.

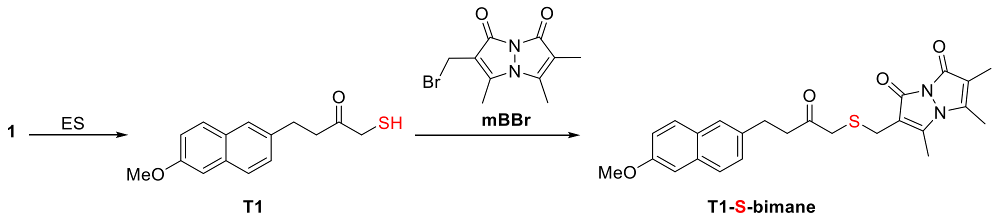

Scheme S2. In the presence of ES, **1** produces **T1** and in the presence of mono-bromobimane (mBBr), produces **T1-S-bimane** (expected, m/z =450.1613, see Fig S3).

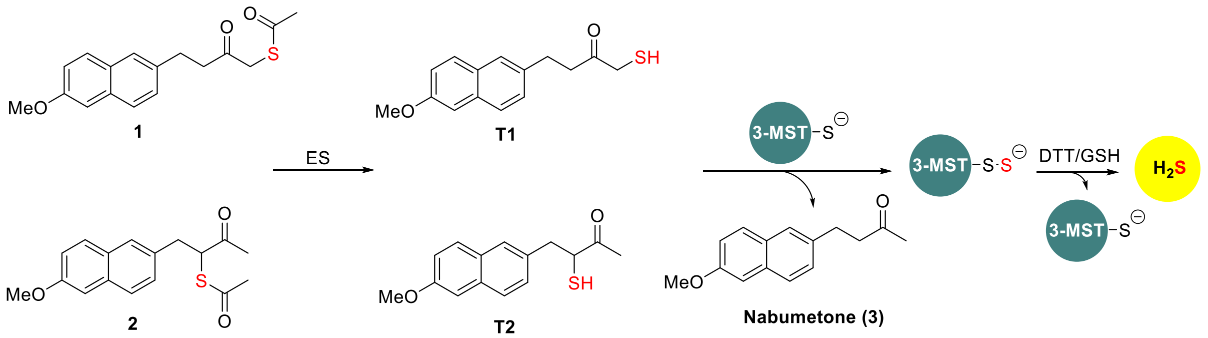

Scheme S3. Reaction of **1** or **2** in the presence of ES gives **T1** or **T2.** Turnover by 3-MST in the presence of reducing conditions (DTT or GSH) produces Nabumetone (**3**).

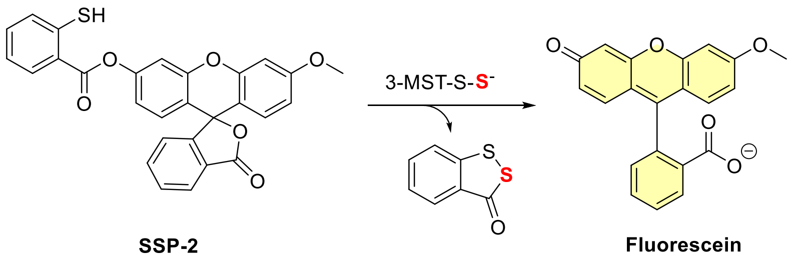

Scheme S4. Detection of persulfide by SSP-2.

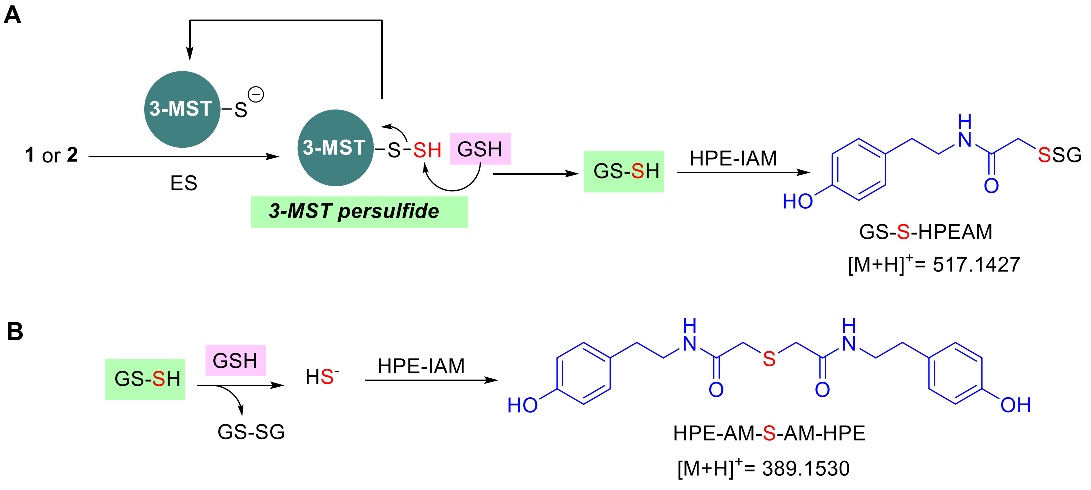

Scheme S5. (**A**) A schematic diagram of the various reactive sulfur species formed during the reaction of ES, 3-MST, and **1** or **2** in the presence of GSH in pH 7.4 1x PBS. GSSH is trapped by the electrophilic reagent HPE-IAM. (**B**) GSSH can produce H_2S to give HPE-AM-S-AM-HPE.

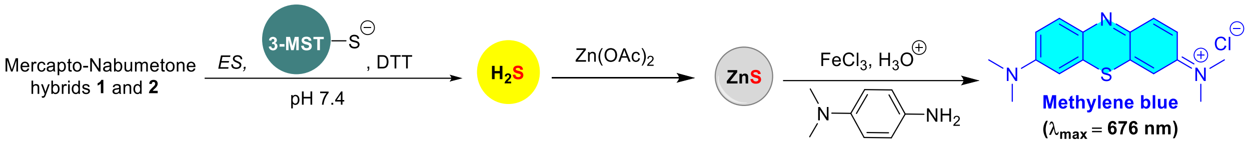

Scheme S6. Methylene blue assay protocol for studying the generation of H_2S from **1** and **2**.

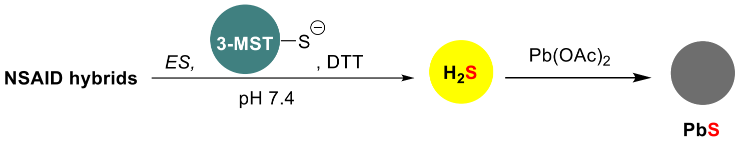

Scheme S7. Lead acetate assay for the detection of H_2S.

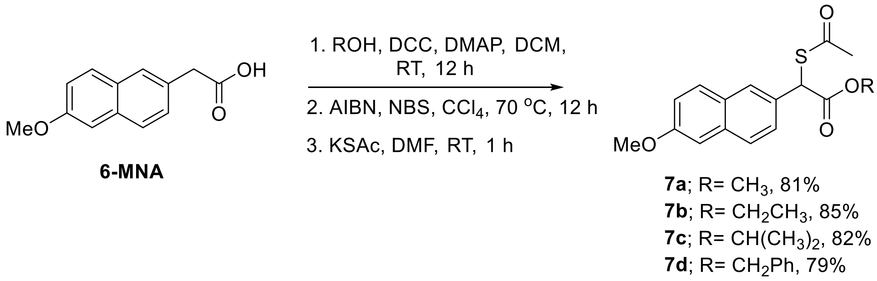

Scheme S8. Synthesis of **7a-7d**.

5. Supplementary Tables

Table S1: Docking scores of thiols in the active site of *h*3-MST. A snapshot of the lowest energy conformation and the S---S distance between the cysteine sulfur and the thiol is shown below.

Structure	Comp. No.	Docking energy (kcal/mol)	CysS-ThiolS (Å)	Lowest energy conformation
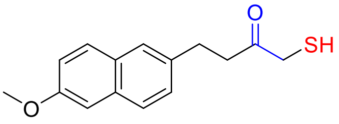	**T1**	-5.5	4.6	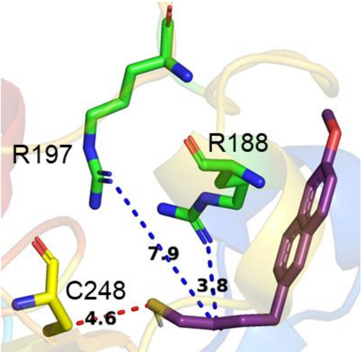
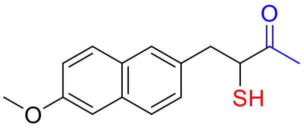	**T2**	-5.3	10.3	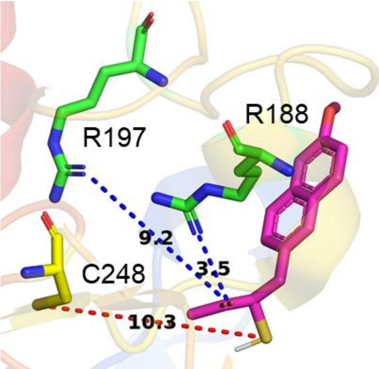
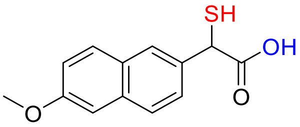	**T3**	-5.4	10.2	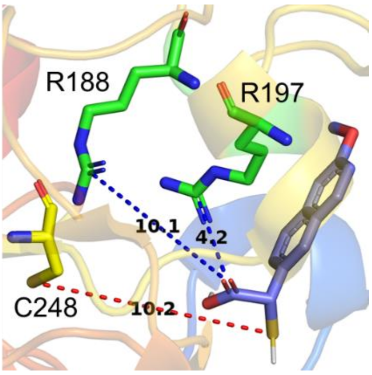

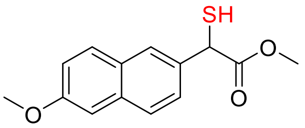	**T7a**	-5.5	10.2	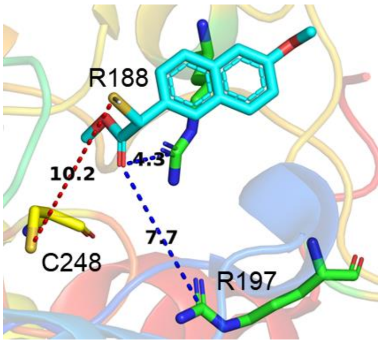
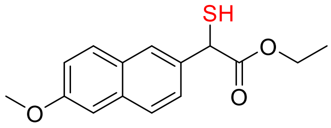	**T7b**	-5.6	10.1	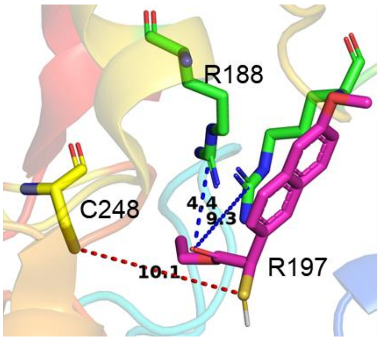
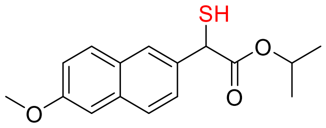	**T7c**	-5.7	10.1	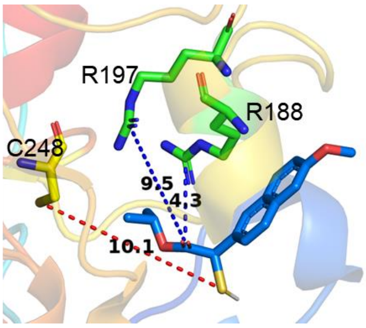
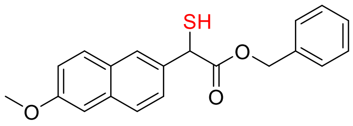	**T7d**	-7.0	10.4	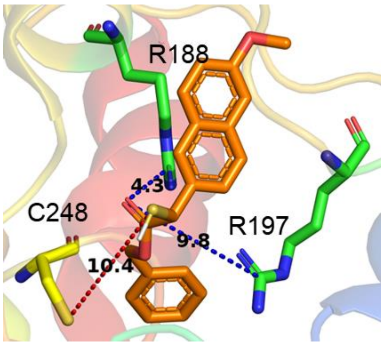

Table S2: Physicochemical properties of mercapto-Nabumetone derivatives **T1** and **T2** and Nabumetone (**3**) calculated using SwissADME.

Cpd.	MW (g/mol)	nHA	nAHA	F.Csp^3	nRB	nHBA	nHBD	MR	TPSA (A^2)[a]
**T1**	260.35	18	10	0.27	5	2	0	77.96	65.10
**T2**	260.35	18	10	0.27	4	2	0	77.96	65.10
**3**	228.29	17	10	0.21	4	2	0	70.03	26.30

[a]The total polar surface area (TPSA) values were higher in the case of the mercapto-NSAIDs, but they were much below the range (140 Å^2) where permeability is expected to be significantly reduced.

Table S3: Lipophilicity of compounds calculated using SwissADME.

Cpd.	iLOGP	XLOGP$_3$	WLOGP	MLOGP	SILICOS IT	Consensus log P
**1**	3.08	3.54	3.63	2.43	4.49	3.43
**2**	2.83	3.64	3.63	2.43	4.32	3.37
**T1**	2.67	3.40	3.28	2.90	4.21	3.29
**T2**	2.41	3.50	3.28	2.90	4.04	3.23
**3**	2.74	3.08	3.37	2.90	4.06	3.23

6. Supplementary figures

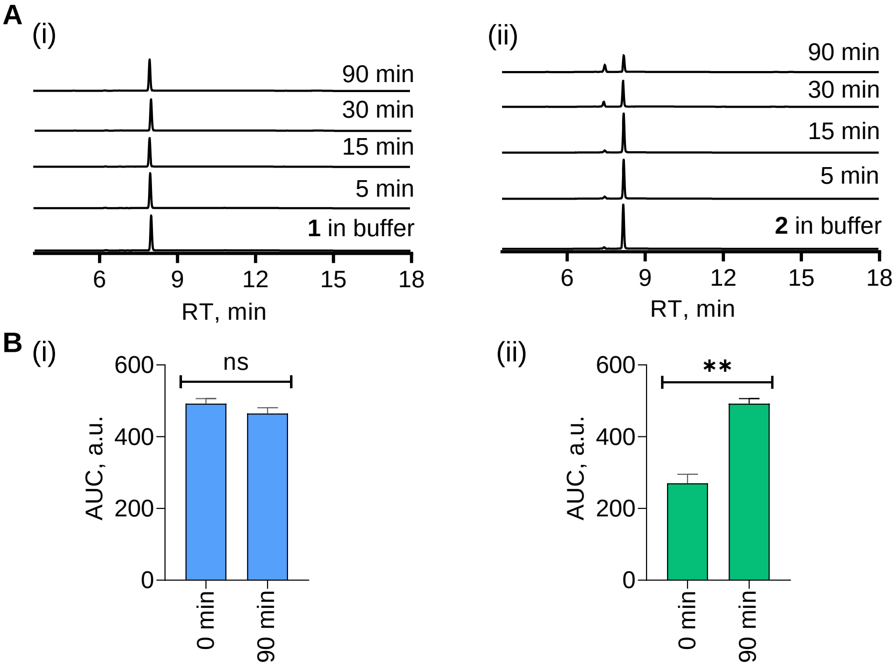

Figure S1. (**A**) HPLC traces of **(i) 1** and **(ii) 2** incubated in PBS pH 7.4 buffer up to 90 min (Abs = 280 nm). (**B**) The AUC values **(i) 1** and **(ii) 2** are plotted. About 54% of compound **2** remained during 90 min. The formation of **T2** was observed, suggesting that the thioacetate **2** was labile under these conditions. All data are represented as mean ± SD (n = 2 per group). Student's two-tailed unpaired parametric t-test was carried out to determine significance (***p* < 0.01 and ns indicates non-significant, Abs = 280 nm).

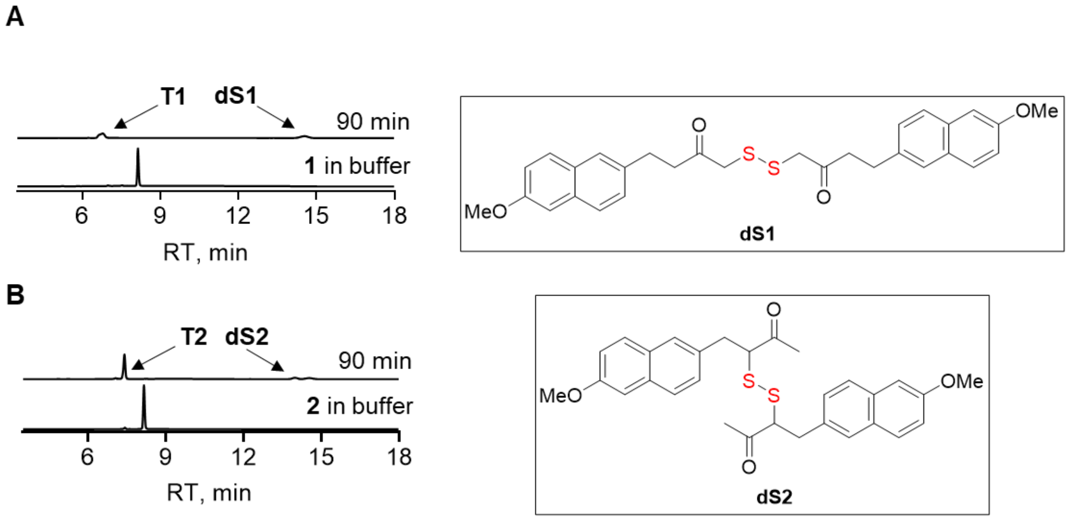

Figure S2. HPLC traces for the conversion of (**A**) **1** to **T1**; and (**B**) **2** to **T2** during incubation with ES in 1x PBS pH 7.4 buffer. Formation of **dS1** and **dS2** due to aerial oxidation of **T1** and **T2** at a retention time of 14.6 and 14.9 min, respectively, was observed (Abs = 280 nm). Note: Under ambient aerobic conditions, significant broadening of the peak corresponding to **T1** was seen.

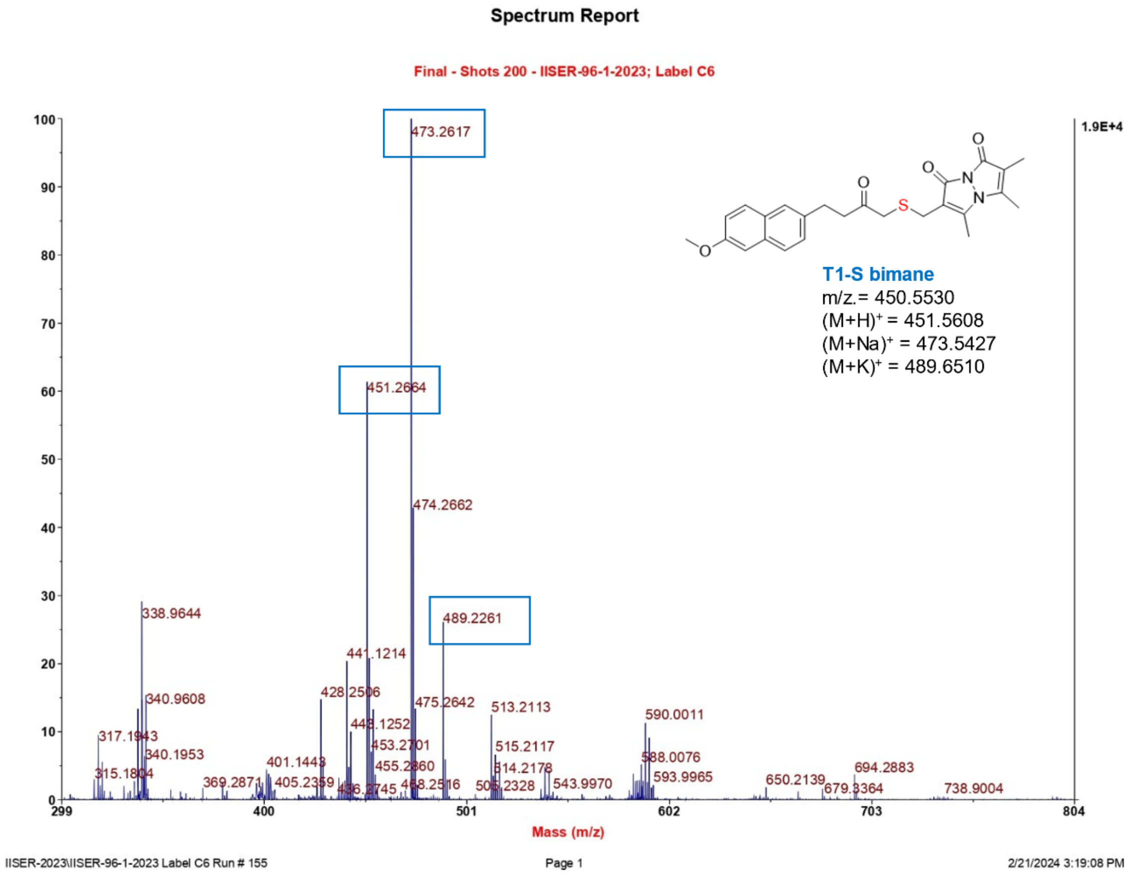

Figure S3. Mass of the **T1-S-bimane** adduct formation when **1** incubated with ES and mBBr. Mass of adducts observed; $[M+H]^+$ = 451.2664, $[M+Na]^+$ 473.2617 $[M+K]^+$ = 489.2261.

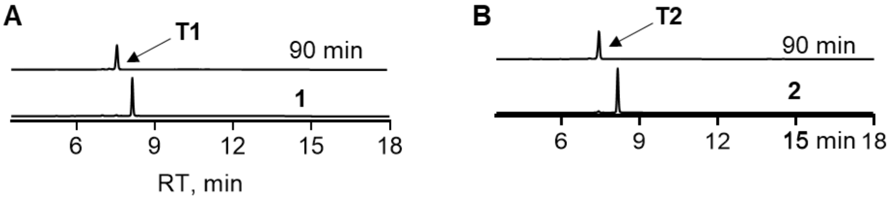

Figure S4. HPLC traces for the conversion of (**A**) **1** to **T1**; and (**B**) **2** to **T2** during incubation with ES and DTT in 1x PBS pH 7.4 buffer, respectively (Abs = 280 nm).

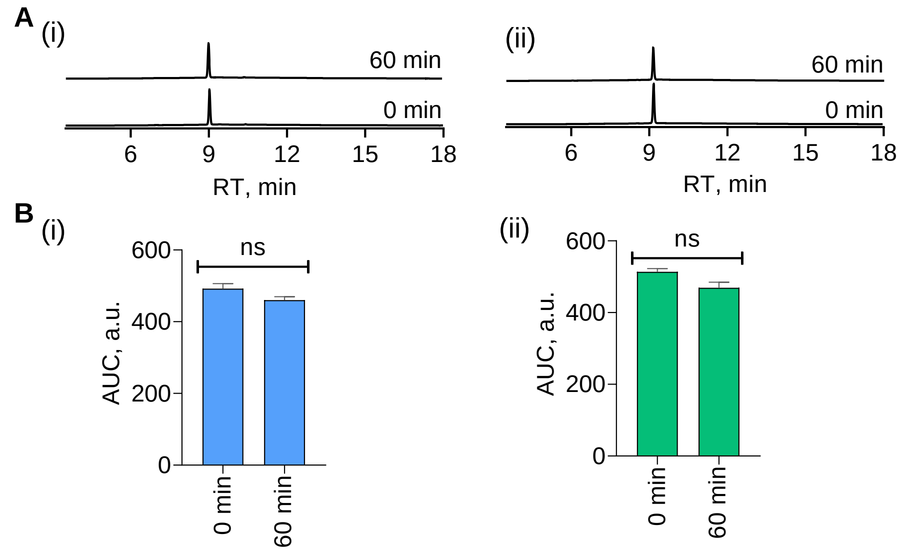

Figure S5. (**A**) HPLC traces of (**i**) **1** and **(ii) 2** incubated in PBS pH 2.0 buffer up to 60 min (Abs = 280 nm). (**B**) The AUC values **(i) 1** and **(ii) 2** are plotted. All data are represented as mean ± SD (n = 2 per group). Student's two-tailed unpaired parametric t-test was carried out to determine significance (ns indicates non-significant, Abs = 280 nm).

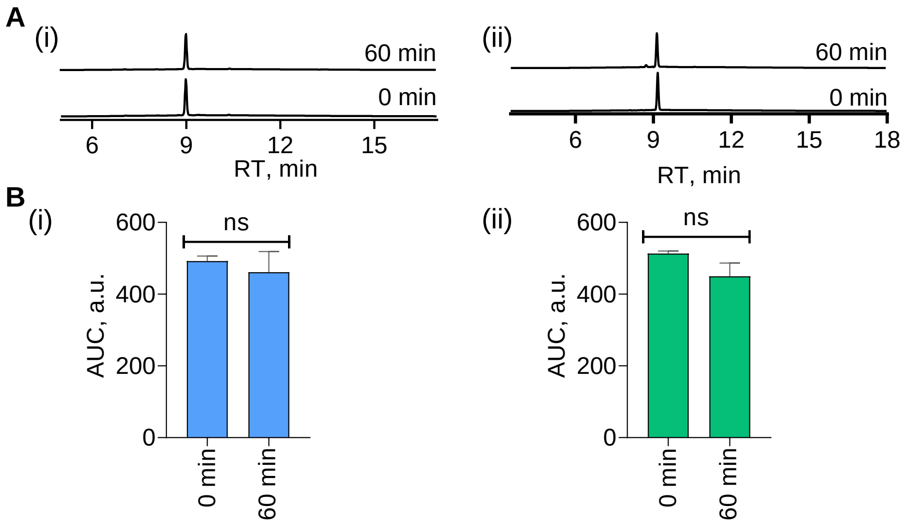

Figure S6. (**A**) HPLC traces of (**i**) **1** and **(ii) 2** incubated in PBS pH 6.8 buffer up to 60 min (Abs = 280 nm). (**B**) The AUC values **(i) 1** and **(ii) 2** are plotted. All data are represented as mean ± SD (n = 2 per group). Student's two-tailed unpaired parametric t-test was carried out to determine significance (ns indicates non-significant, Abs = 280 nm).

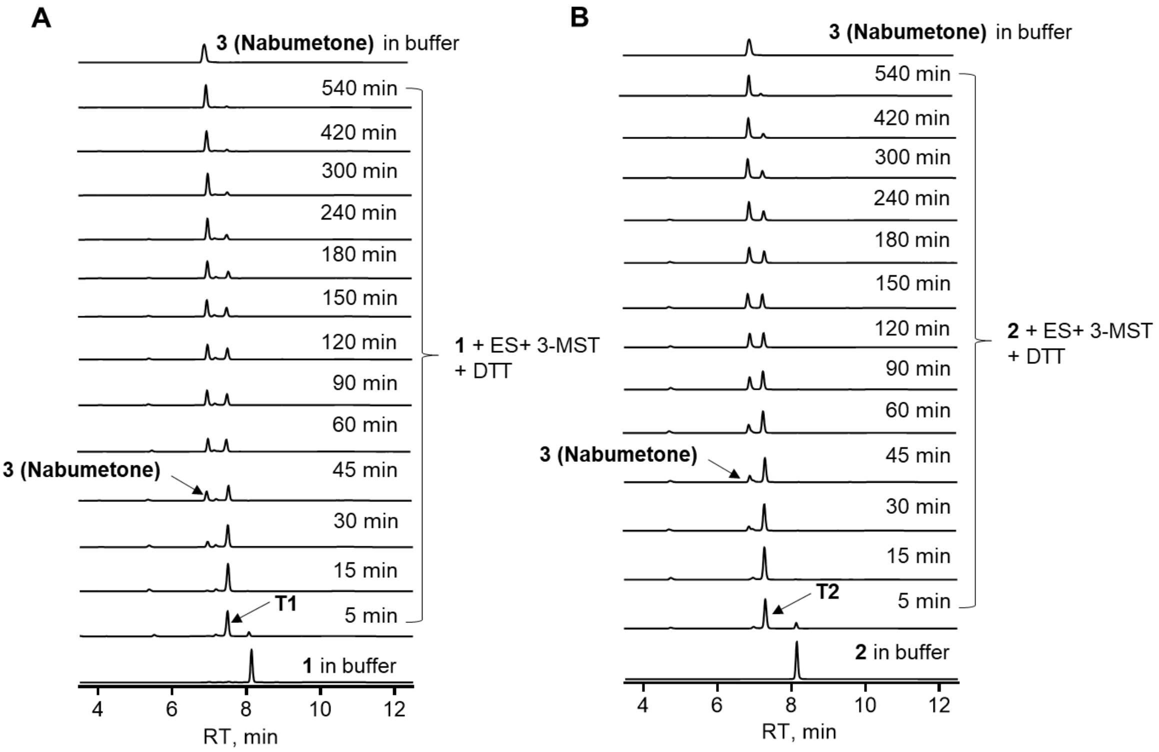

Figure S7. Representative HPLC traces for the conversion of (**A**) **1** to **T1**, turnover of **T1** to Nabumetone, (B) **2** to **T2**, and turnover of **T2** to Nabumetone. Reaction conditions: ES, 3-MST, and DTT in 1x PBS pH 7.4 buffer (Abs = 280 nm).

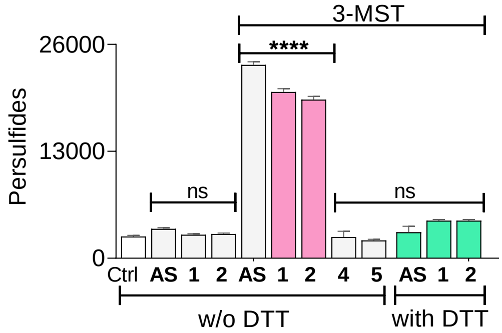

Figure S8. Persulfide/polysulfide detection for **1**, **2**, **4**, and **5** using probe SSP-2 (Fluorescence intensities were measured; λ_{ex} 482 nm, λ_{em} 518 nm). DTT refers to the post-treatment of the reaction with DTT to reduce 3-MST persulfides and release H_2S, which shows no fluorescein signal. **AS** was used as a positive control. Ctrl represents only 3-MST. For statistical analysis, the student's two-tailed unpaired parametric *t*-test was used to determine significance: ns = non-significant, ****$p < 0.0001$ with respect to 3-MST only. All the results are represented as mean ± SD (n = 3 per group). SD is standard deviation.

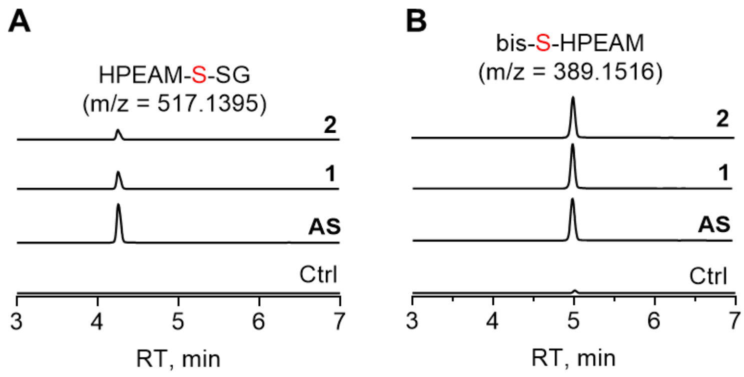

Figure S9. Extracted ion chromatograms from an LC-MS/MS analysis of (**A**) HPEAM-S-SG (expected, $[M+H]^+ = 517.1427$; observed $[M+H]^+ = 517.1395$), and (**B**) bis-S-HPE-AM (expected, $[M+H]^+ = 389.1530$; observed $[M+H]^+ = 389.1516$) indicating the formation of GSSH and H_2S, upon reacting **AS**, **1,** and **2** with 3-MST in the presence of ES, followed by sequential addition of thiol acceptor GSH and electrophilic trapping reagent HPE-IAM. Ctrl represents only 3-MST.

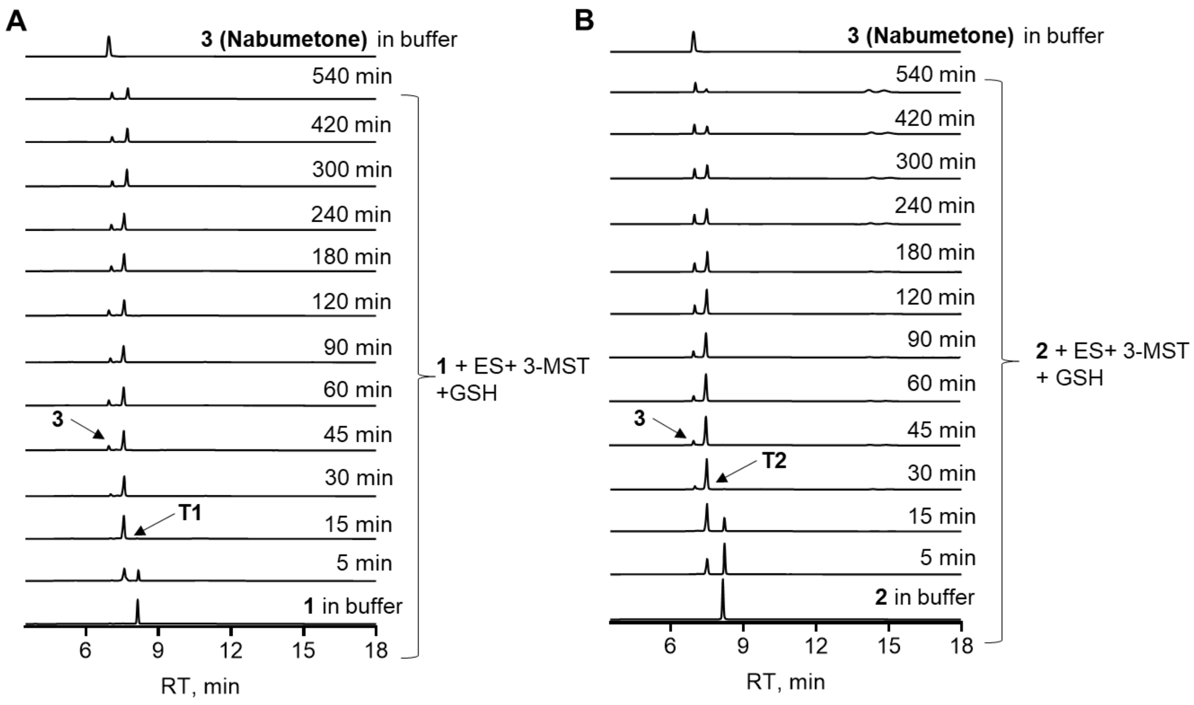

Figure S10. Representative HPLC traces for decomposition of (**A**) **1** to **T1**, turnover of **T1** to Nabumetone, (**B**) **2** to **T2**, and turnover of **T2** to Nabumetone. Reaction conditions: ES, 3-MST, and GSH in 1x PBS pH 7.4 buffer (Abs = 280 nm).

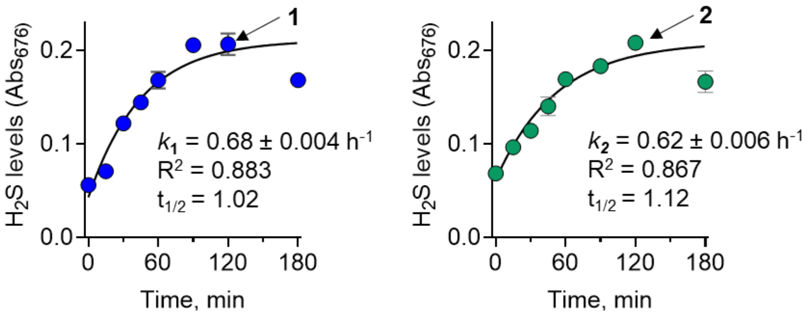

Figure S11. Detection of H_2S using methylene blue assay for **1** or **2** in the presence of ES, 3-MST, and DTT was done at various time intervals. Curve fitting using a pseudo-first-order equation $y = y^0 + a\,(1 - e^{-kx})$ was carried out and gave the values listed. All data are presented as mean ± SD (n = 3 per group).

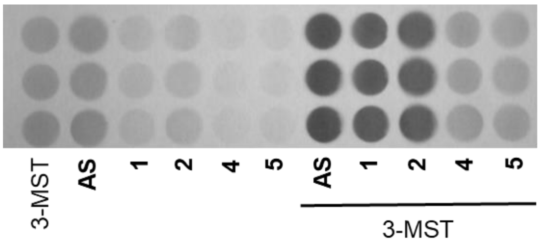

Figure S12. H_2S detection from **AS, 1, 2, 4**, or **5** in the presence of ES, 3-MST, and DTT using a lead acetate assay.

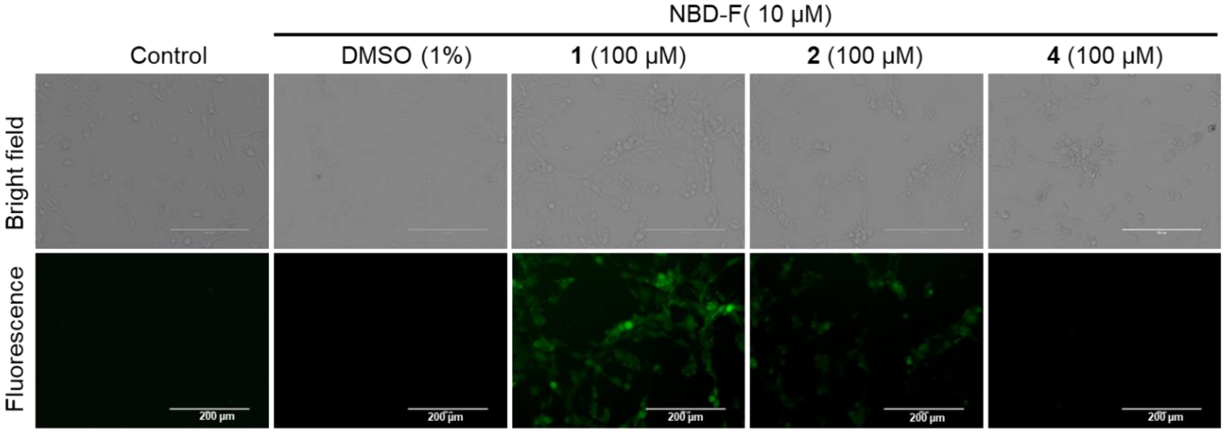

Figure S13. H_2S detection using the NDB-Fluorescein dye in MEF cells. The cells were imaged under the 20x GFP filter. Scale bar is 200 µm

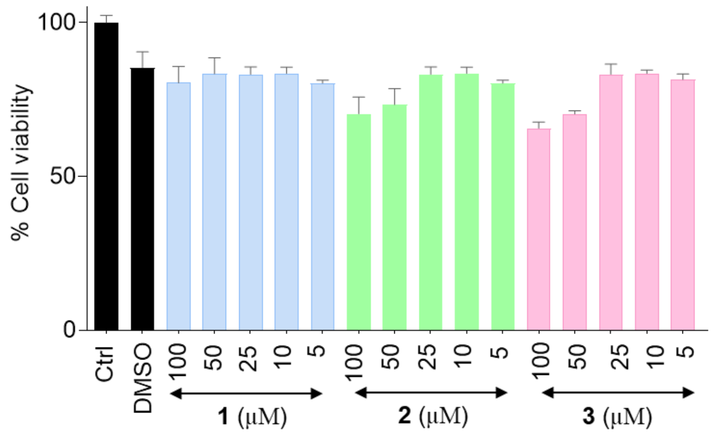

Figure S14. Cell viability assay conducted with MEF cells: Cells were treated with **1, 2,** or **3** with varying concentrations. All data are presented as mean ± SD (n=3 per group).

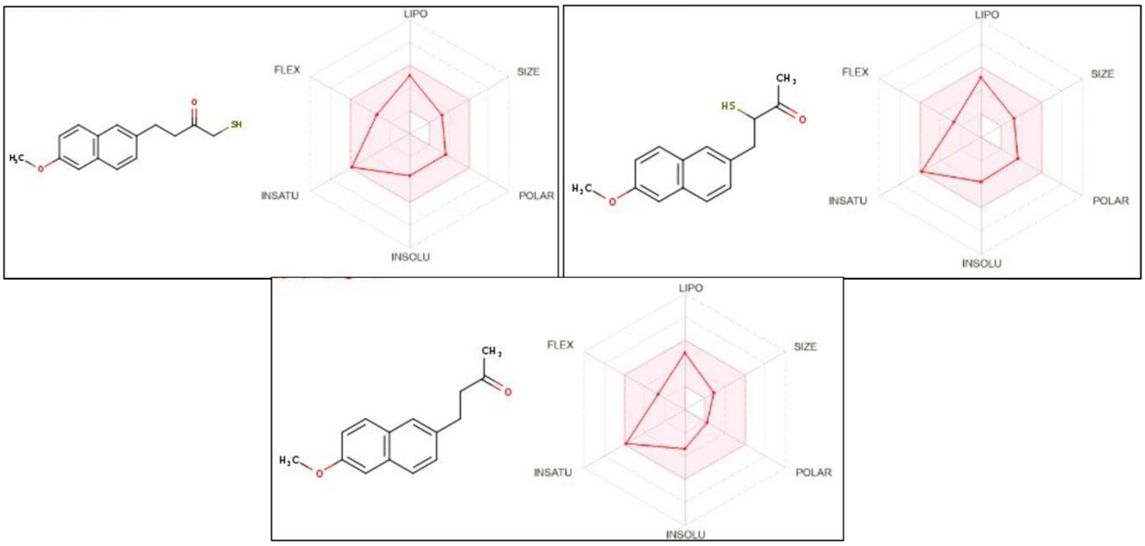

Figure S15. The radar charts of compounds **T1**, **T2**, and **3** describing physicochemical properties calculated by Swiss ADME.

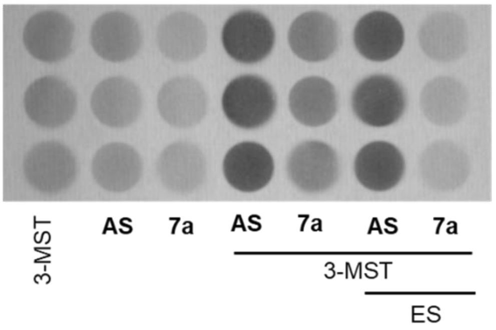

Figure S16. Lead acetate assay for the detection of H_2S under the standard reaction conditions. Compound **7a** produced higher levels of H_2S in the absence of ES.

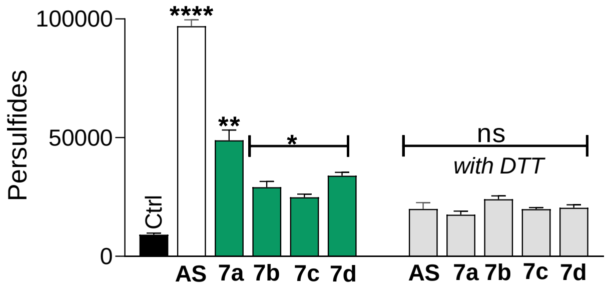

Figure S17. Persulfide/polysulfide detection for **7a - 7d** using probe SSP-2 (Fluorescence intensities were measured; λ_{ex} 482 nm, λ_{em} 518 nm). DTT refers to the post-treatment reaction with DTT to reduce 3-MST persulfides and release H_2S, which shows no fluorescein signal. **AS** was used as a positive control. Ctrl represents only 3-MST. For statistical analysis, the student's two-tailed unpaired parametric *t*-test was used to determine significance: ns = non-significant, ****$p < 0.0001$, **$p < 0.01$, *$p < 0.1$ with respect to 3-MST only. All the results are represented as mean ± SD (n = 3 per group). SD is standard deviation.

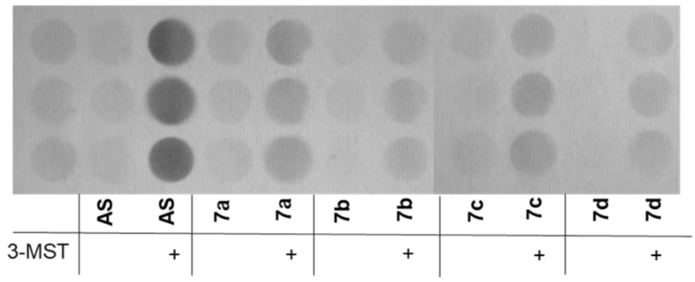

Figure S18. H_2S generation by the 6-MNA thioester substrates **7a - 7d** under the standard reaction conditions. + represents the addition of the 3-MST enzyme.

7. References

1. P. Bora, S. Manna, M. A. Nair, R. R. M. Sathe, S. Singh, V. S. Sreyas Adury, K. Gupta, A. Mukherjee, D. K. Saini, S. S. Kamat, A. B. Hazra, H. Chakrapani, *Chem. Sci*. 2021, **12**, 12939–12949.
2. C. Wei, Q. Zhu, W. Liu, W. Chen, Z. Xi and L. Yi, *Org. Biomol. Chem*., 2014, **12**, 479-485.
3. Liu, C.; Pan, J.; Li, S.; Zhao, Y.; Wu, L. Y.; Berkman, C. E.; Whorton, A. R.; Xian, M, *Angew. Chemie Int. Ed.* 2011, **50**, 10327–10329.
4. Y. Zai, Y. Feng; X. Zeng, X. Tang, Y. Sun, L. Lin, *RSC Adv.* 2019, **9**, 10091–10093.
5. L. Česlová, M. Holčapek and M. Nobilis, *J. Pharm. Biomed. Anal.*, 2014, **88**, 221–224.
6. B. Neises and W. Steglich, *Angew. Chemie Int. Ed. English*, 1978, **17**, 522–524.
7. L. K. Wadhwa and P. D. Sharma, *Indian J. Chem*., 2004, **43**, 1292–1298.
8. S. Mook, J. Lee, J. Ho, M, Ki, S. Lee, H. Ho, J. Shin,K. Lee,J. Yeol, *Bioorg. Med. Chem.* Lett. 2014, **24**, 5418–5422.
9. M. R. Filipovic, J. Zivanovic, B. Alvarez and R. Banerjee, *Chem. Rev.*, 2018, **118**, 1253–1337.
10. D. Seeliger and B. L. de Groot, *J. Comput. Aided. Mol. Des.*, 2010, **24**, 417–422.
11. W. Chen, C. Liu, B. Peng, Y. Zhao, A. Pacheco and M. Xian, *Chem. Sci*., 2013, **4**, 2892.
12. B. S. Choudhary, T. A. Kumar, A. Vashishtha, S. Tejasri, A. S. Kumar, R. Agarwal and H. Chakrapani, *Chem. Commun.*, 2024, **60**, 1727–1730.
13. D. J. Leggett, N. H. Chen and D. S. Mahadevappa, *Anal. Chim. Acta*, 1981, **128**, 163-168.
14. A. K. Sharma, M. Nair, P. Chauhan, K. Gupta, D. K. Saini and H. Chakrapani, *Org. Lett*., 2017, **19**, 4822–4825.
15. A. Daina, O. Michielin and V. Zoete, *Sci. Rep.*, 2017, **7**, 42717.

8. NMR spectra of compounds:

^{1}H NMR spectra of **6a:**

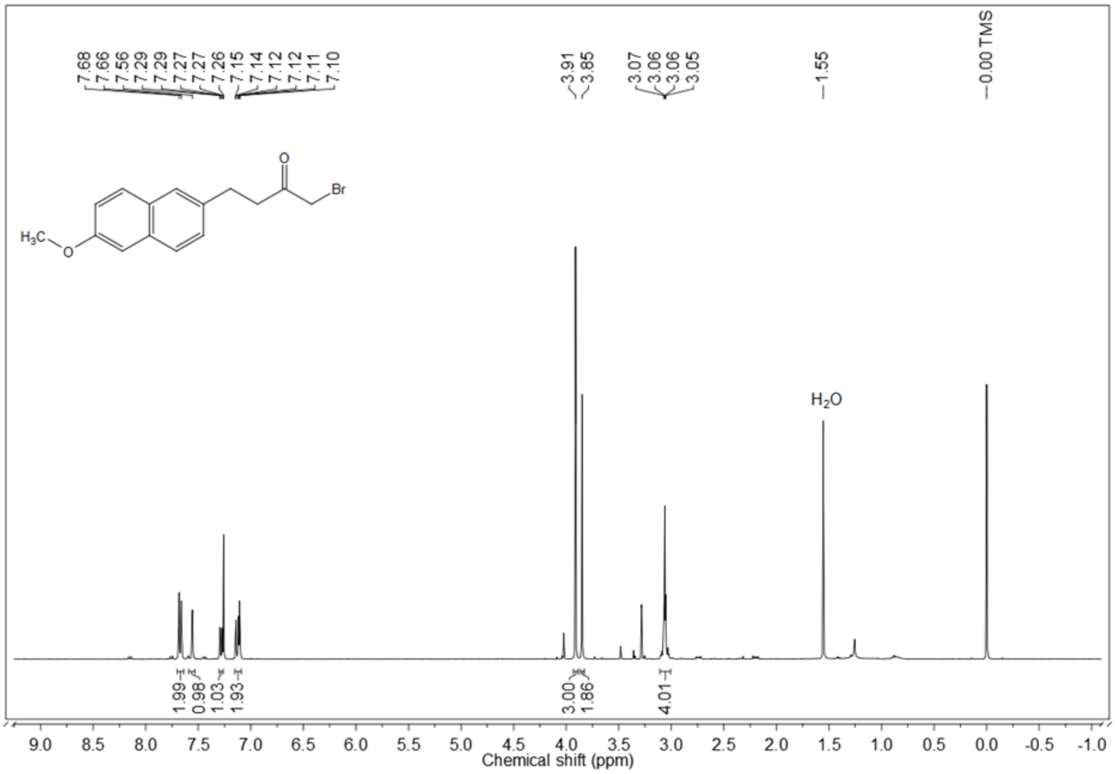

^{13}C NMR spectra of **6a:**

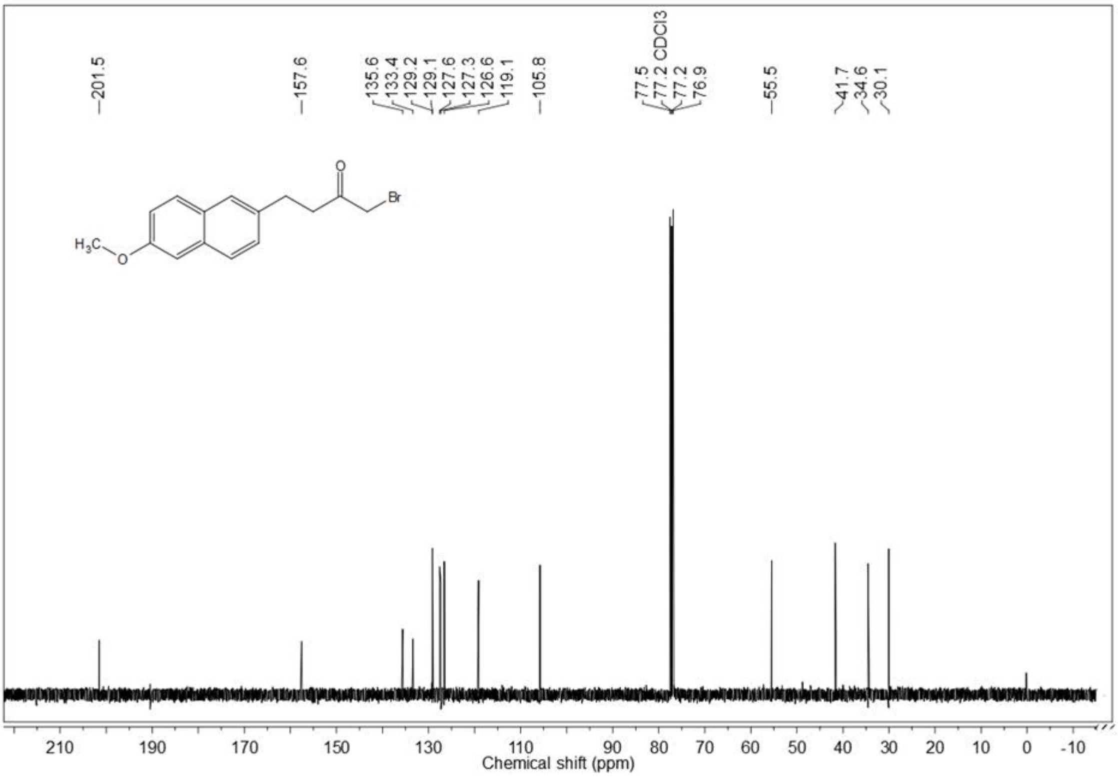

^{1}H NMR spectra of **1:**

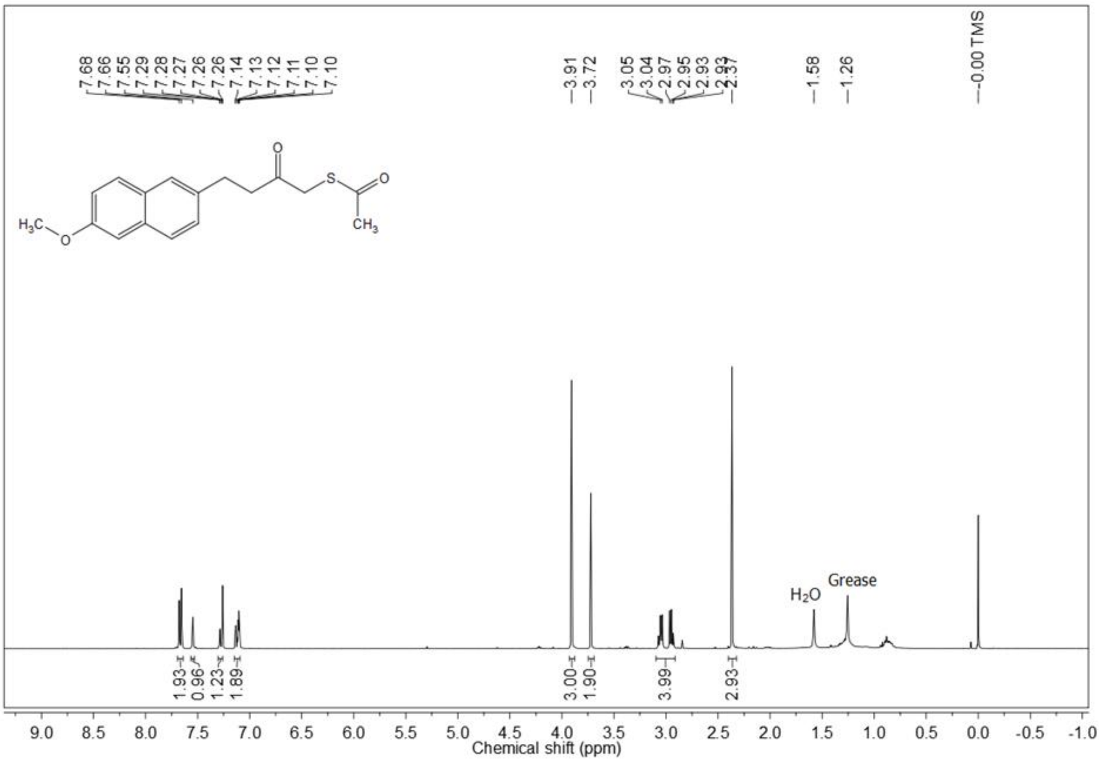

^{13}C NMR spectra of **1:**

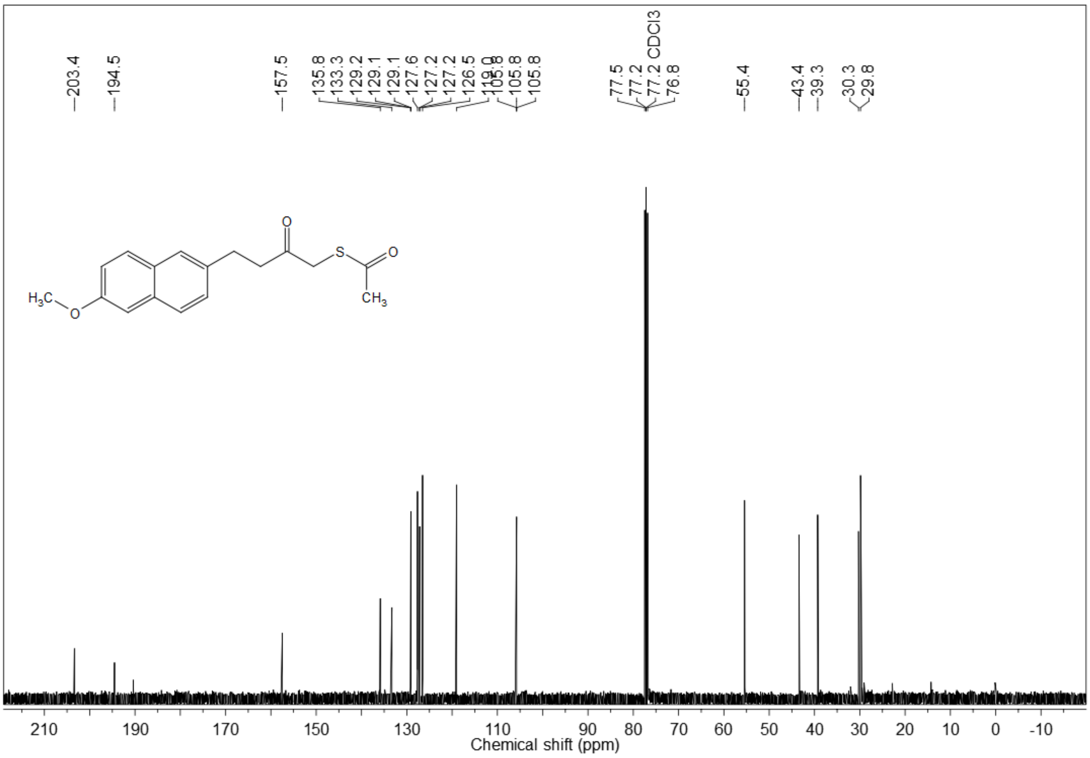

^{1}H NMR spectra of **2:**

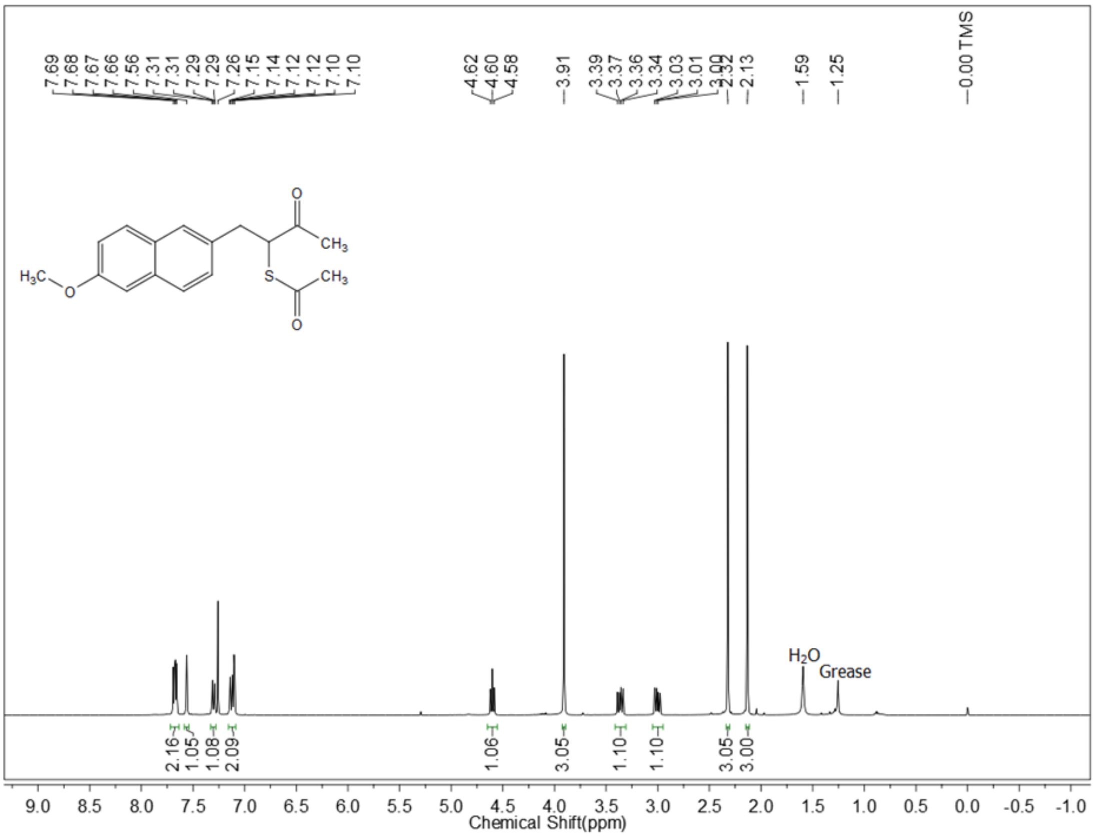

^{13}C NMR spectra of **2:**

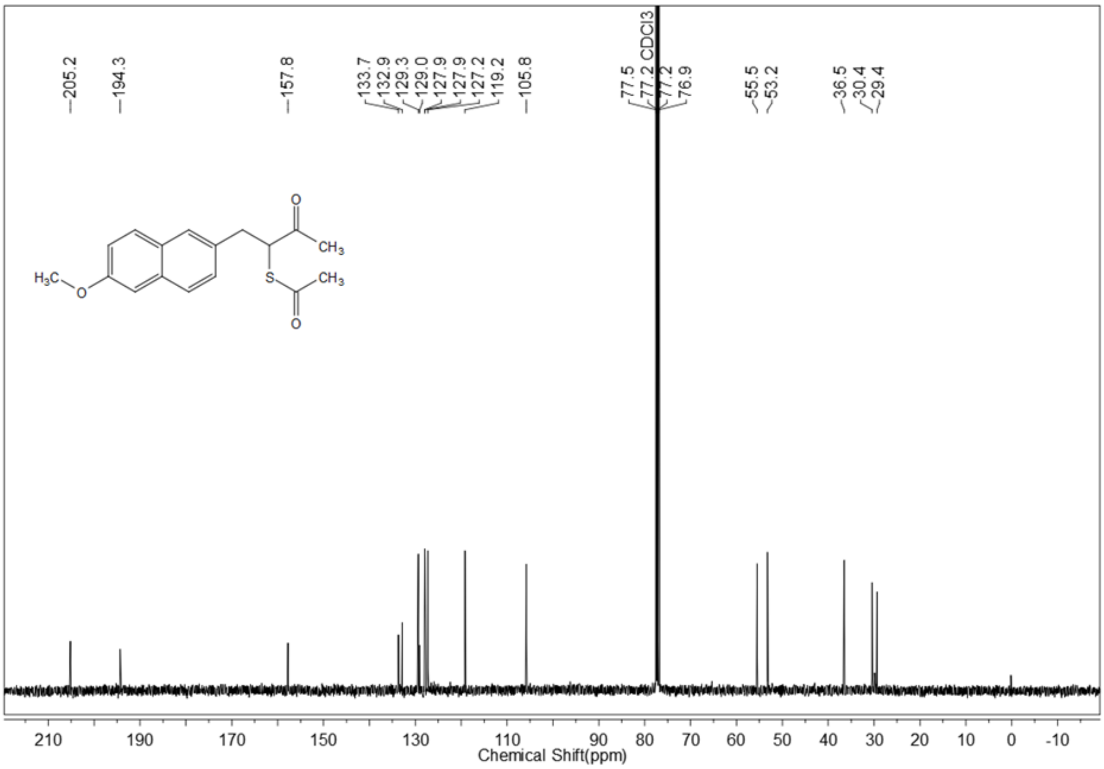

^{1}H NMR spectra of **4:**

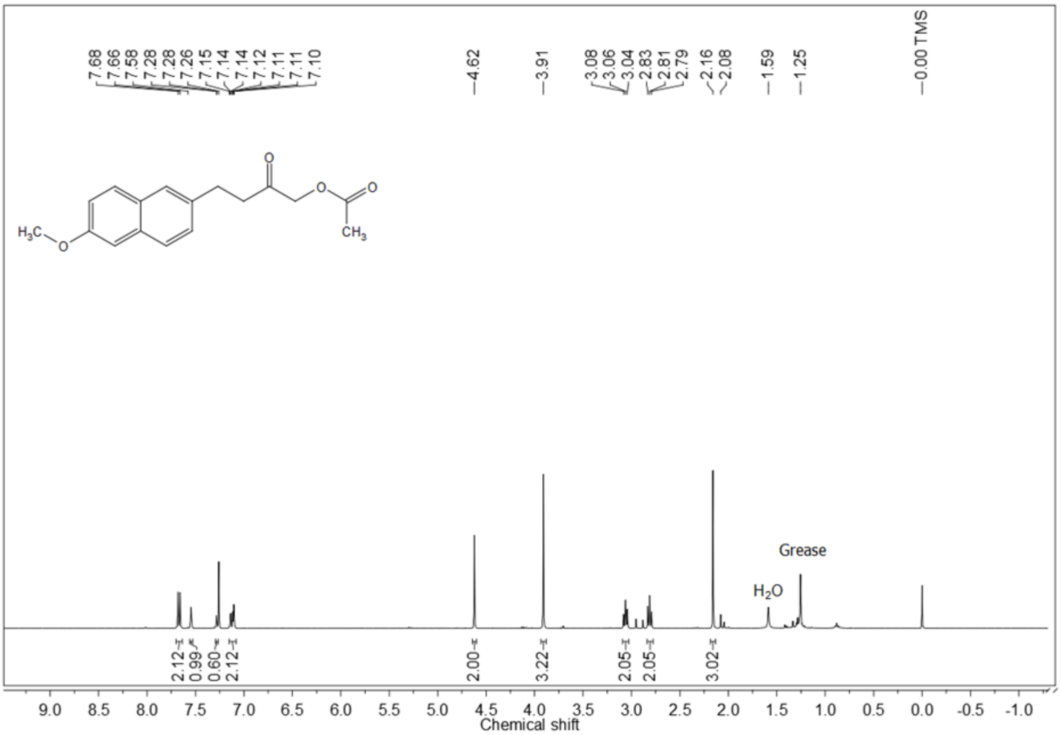

^{13}C NMR spectra of **4:**

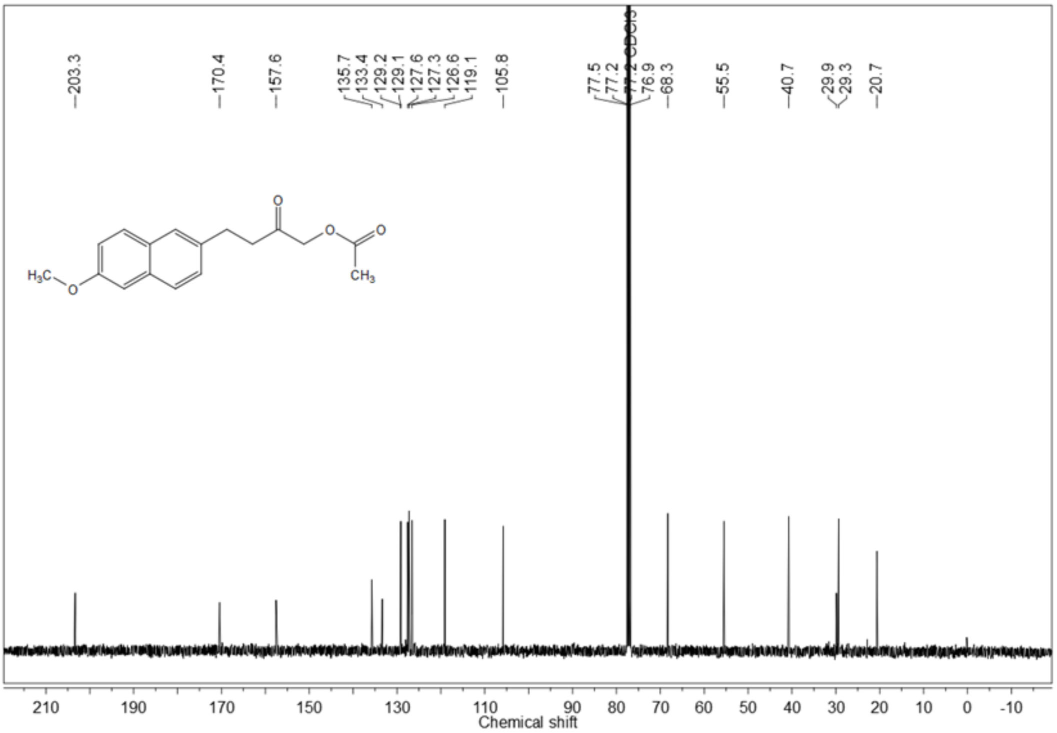

^{1}H NMR spectra of **5:**

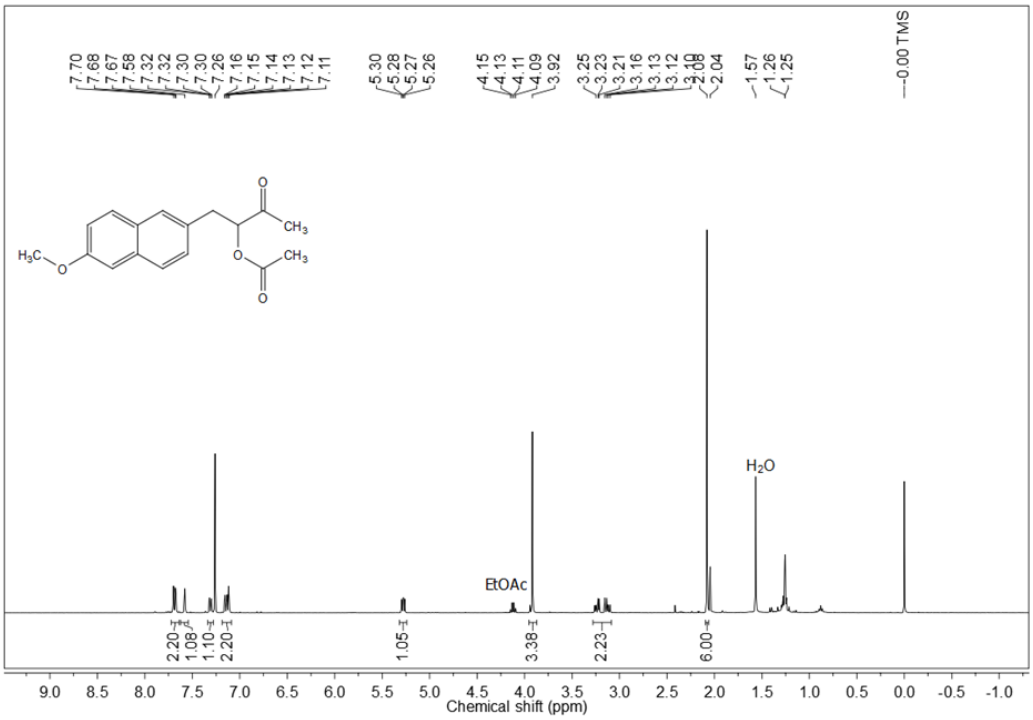

^{13}C NMR spectra of **5:**

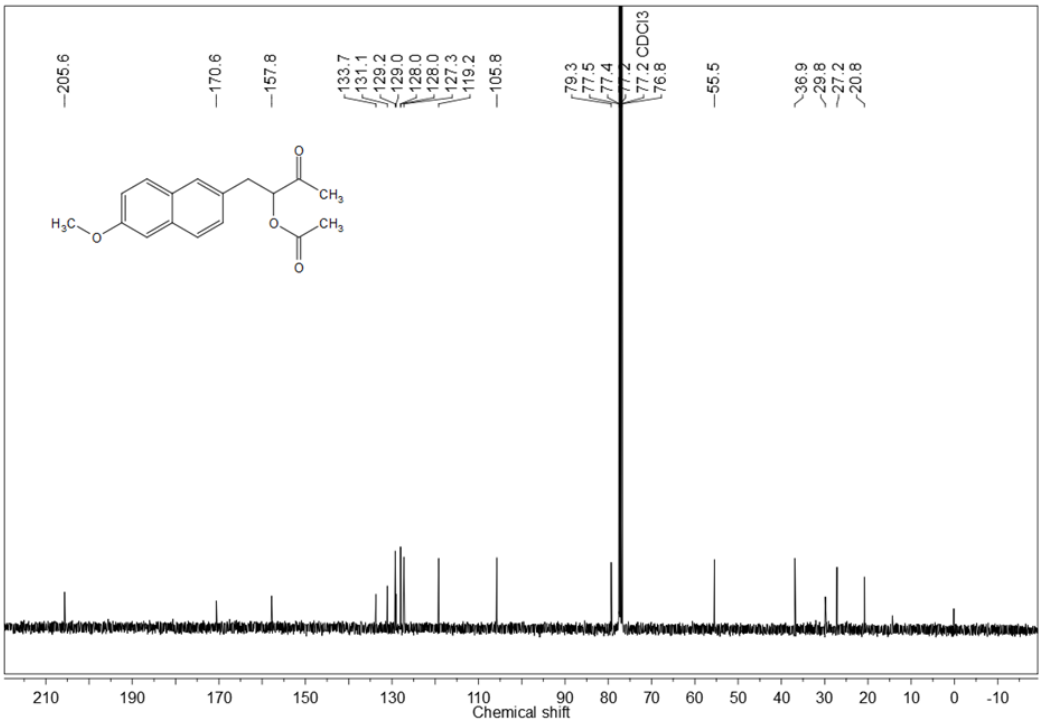

^{1}H NMR spectra of **8a:**

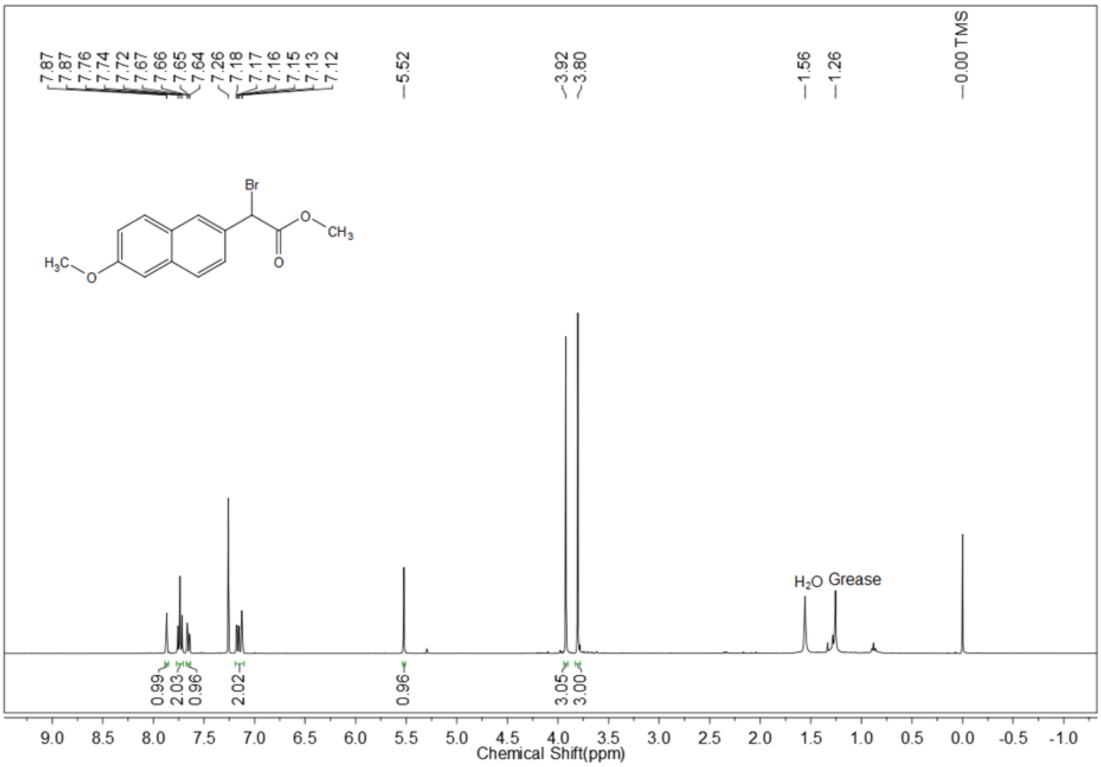

^{13}C NMR spectra of **8a:**

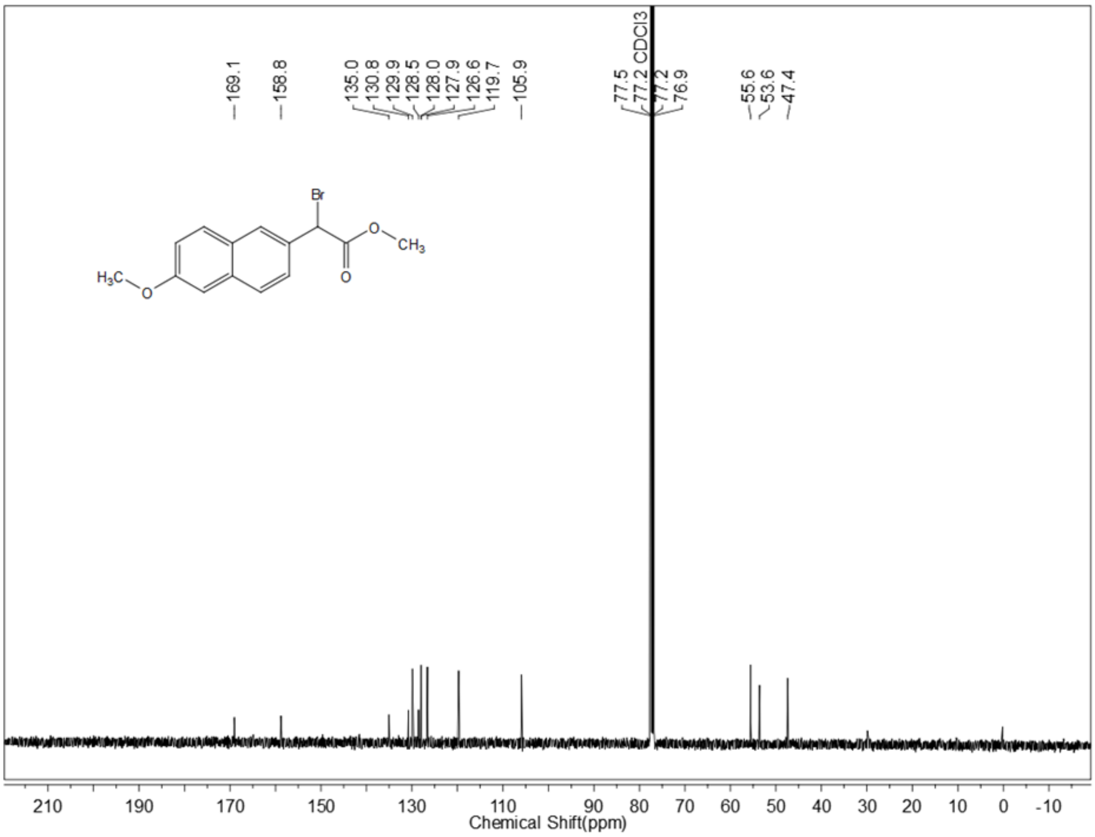

^{1}H NMR spectra of **8b:**

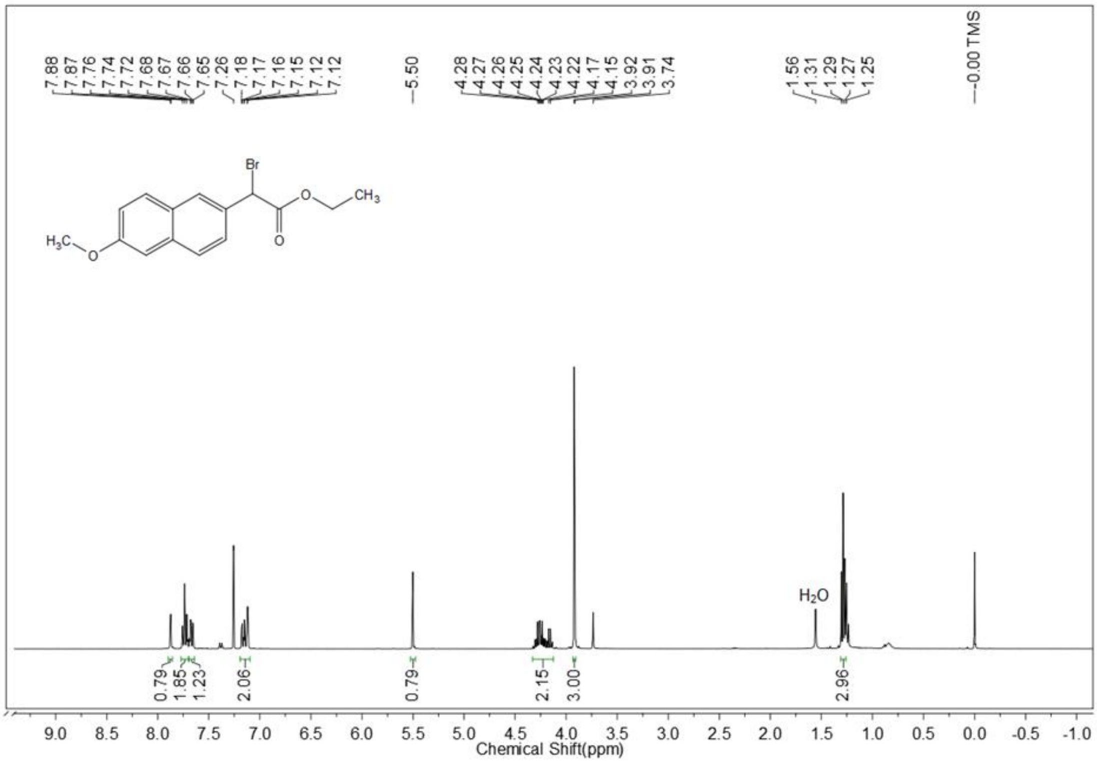

^{13}C NMR spectra of **8b:**

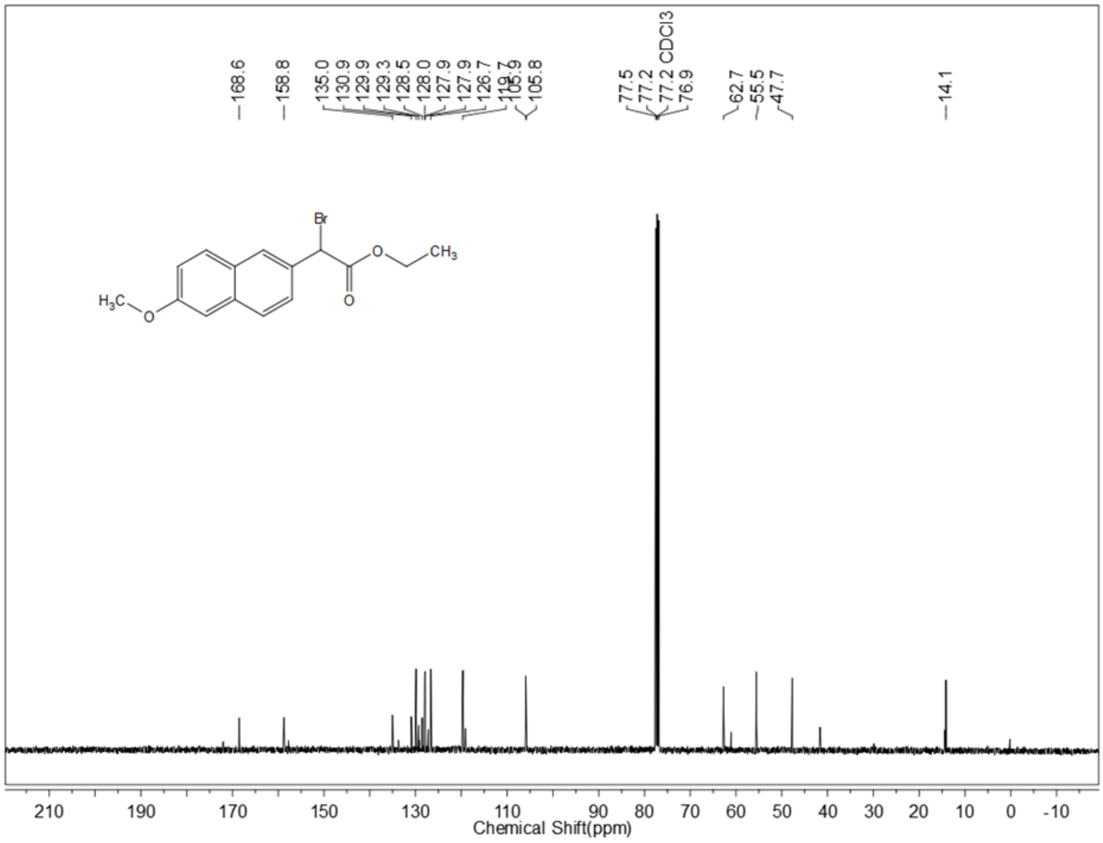

^{1}H NMR spectra of 7a:

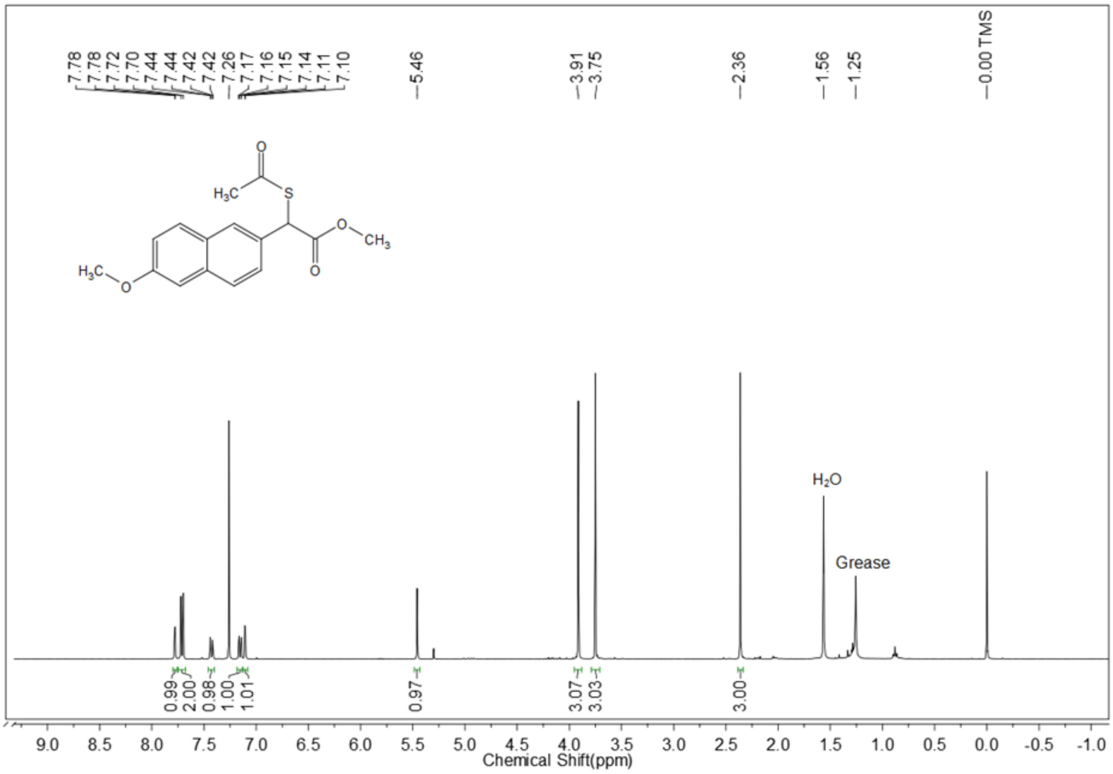

^{13}C NMR spectra of **7a:**

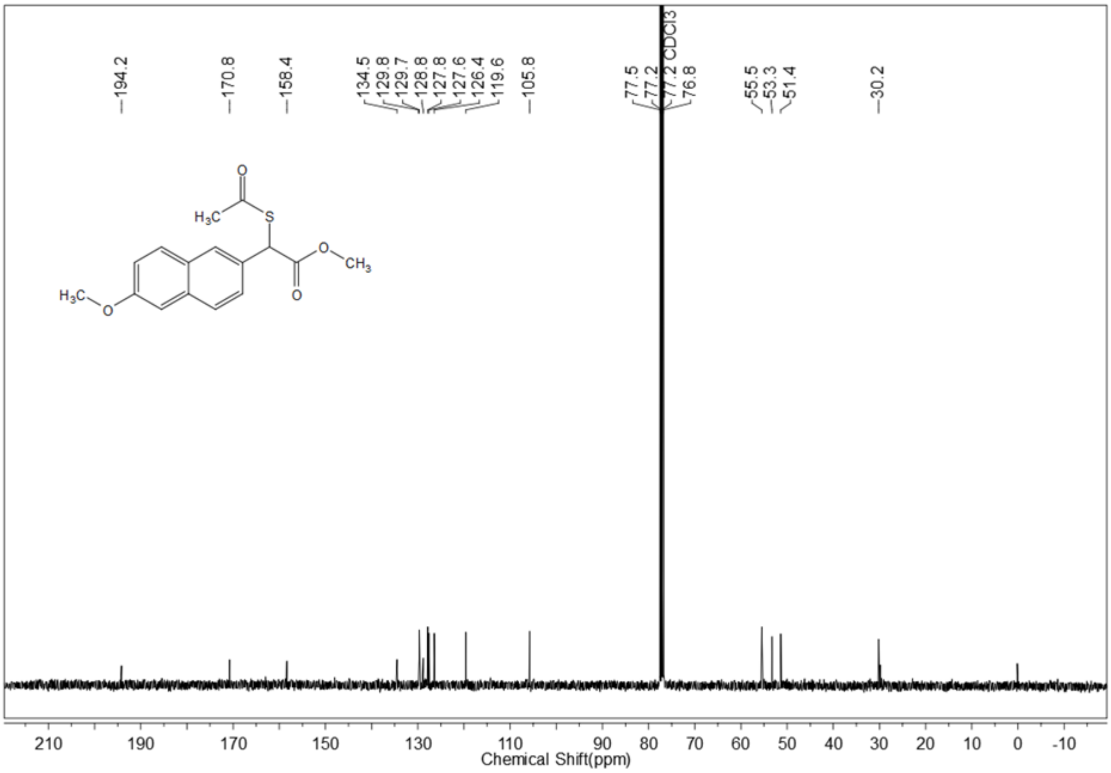

^{1}H NMR spectra of **7b:**

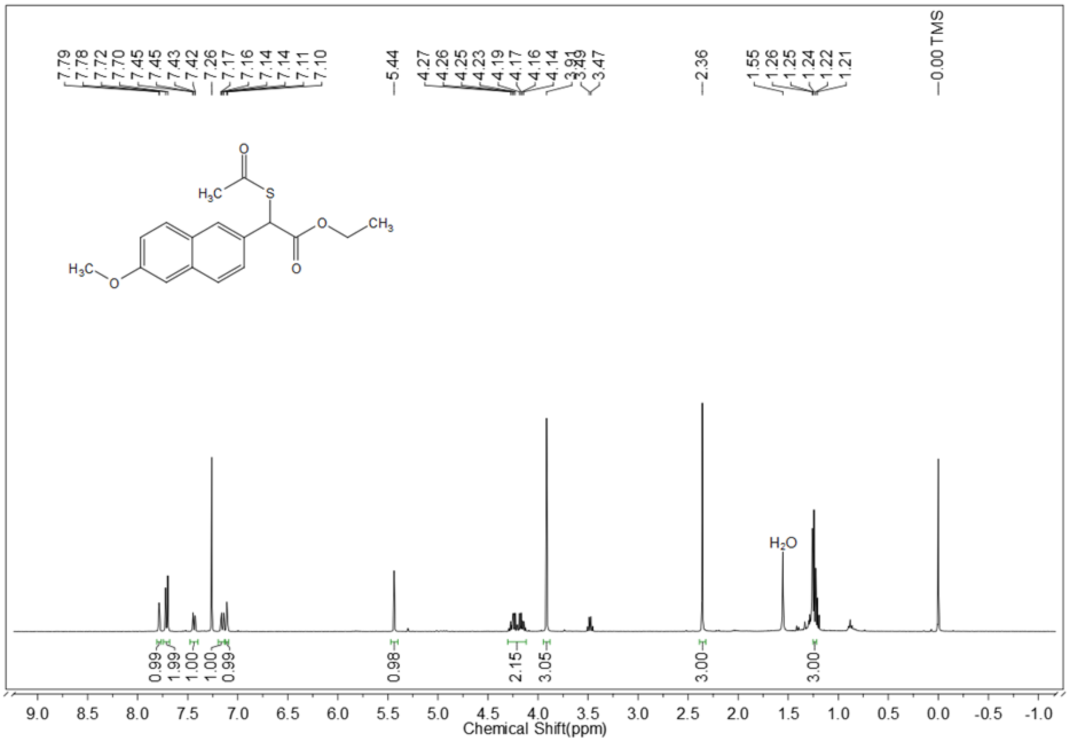

^{13}C NMR spectra of **7b:**

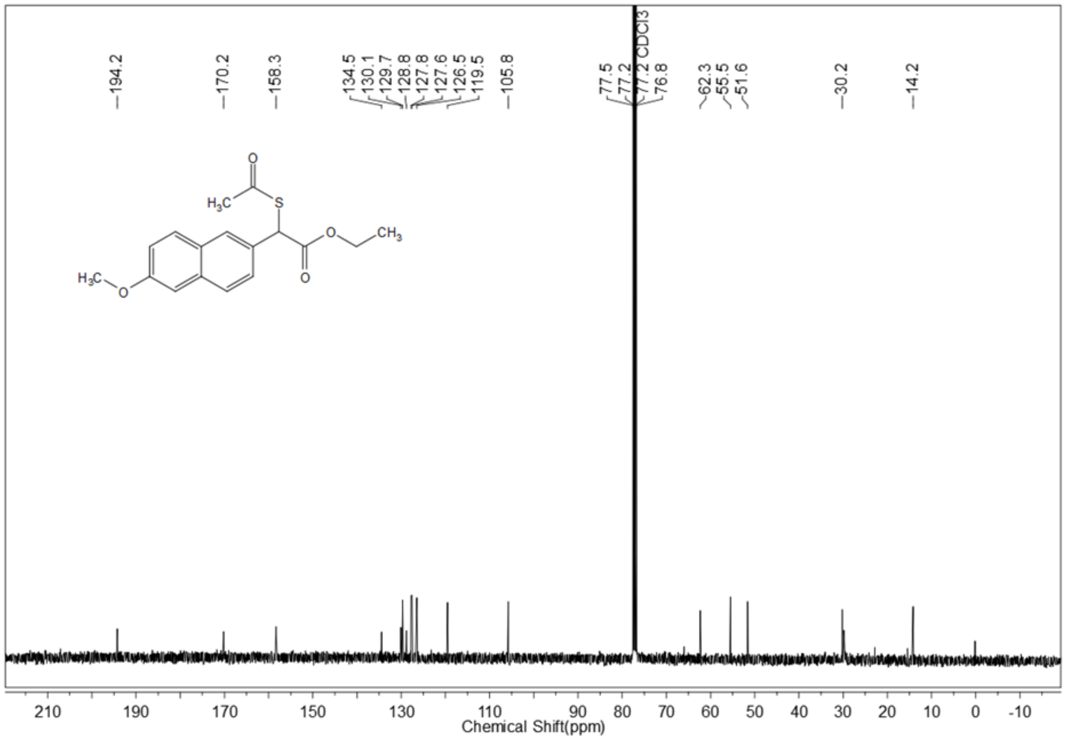

^{1}H NMR spectra of **7c**:

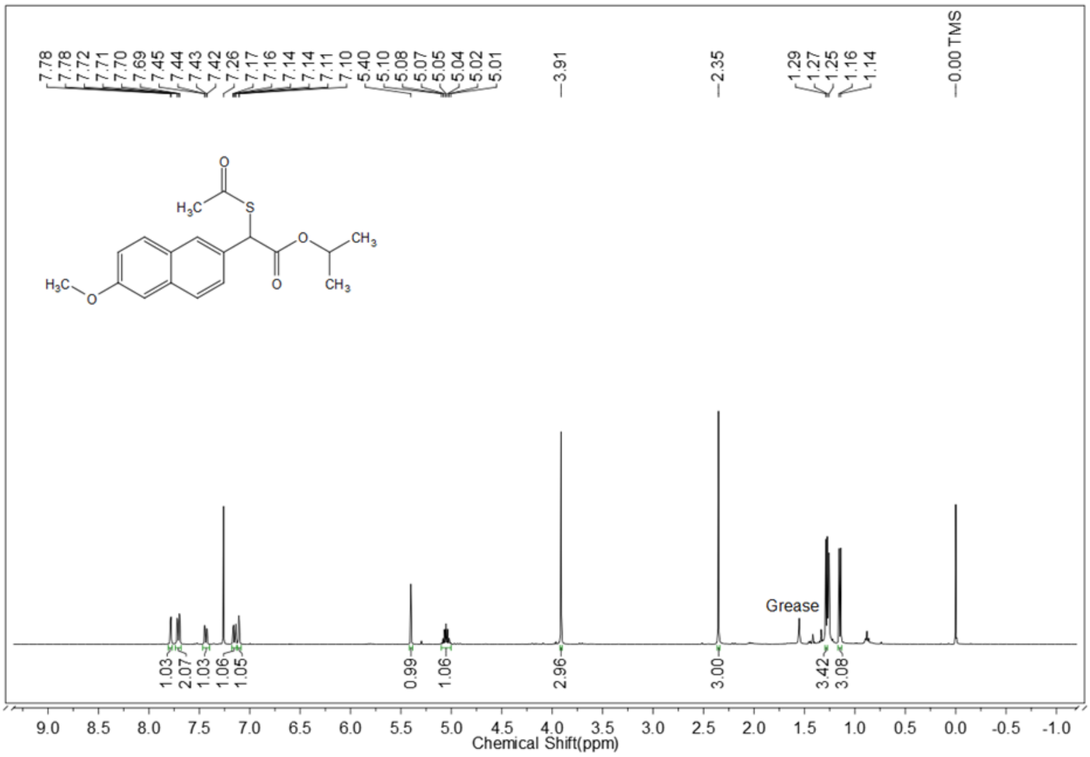

^{13}C NMR spectra of **7c**:

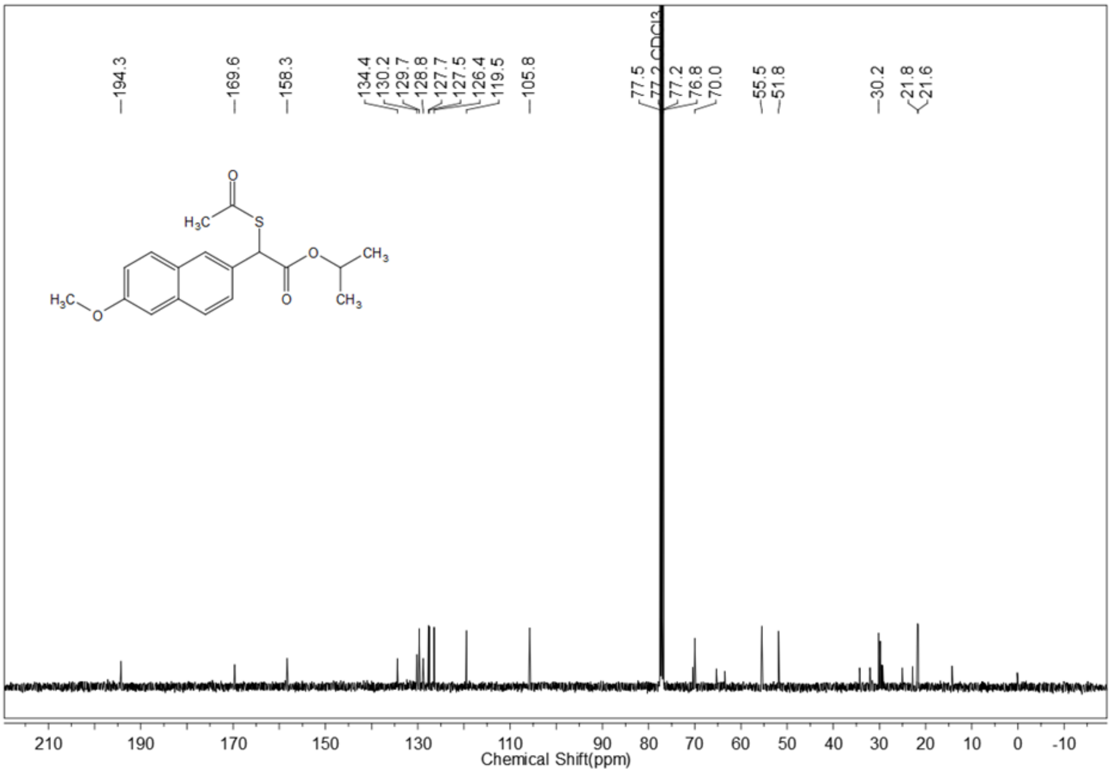

^{1}H NMR spectra of **7d**:

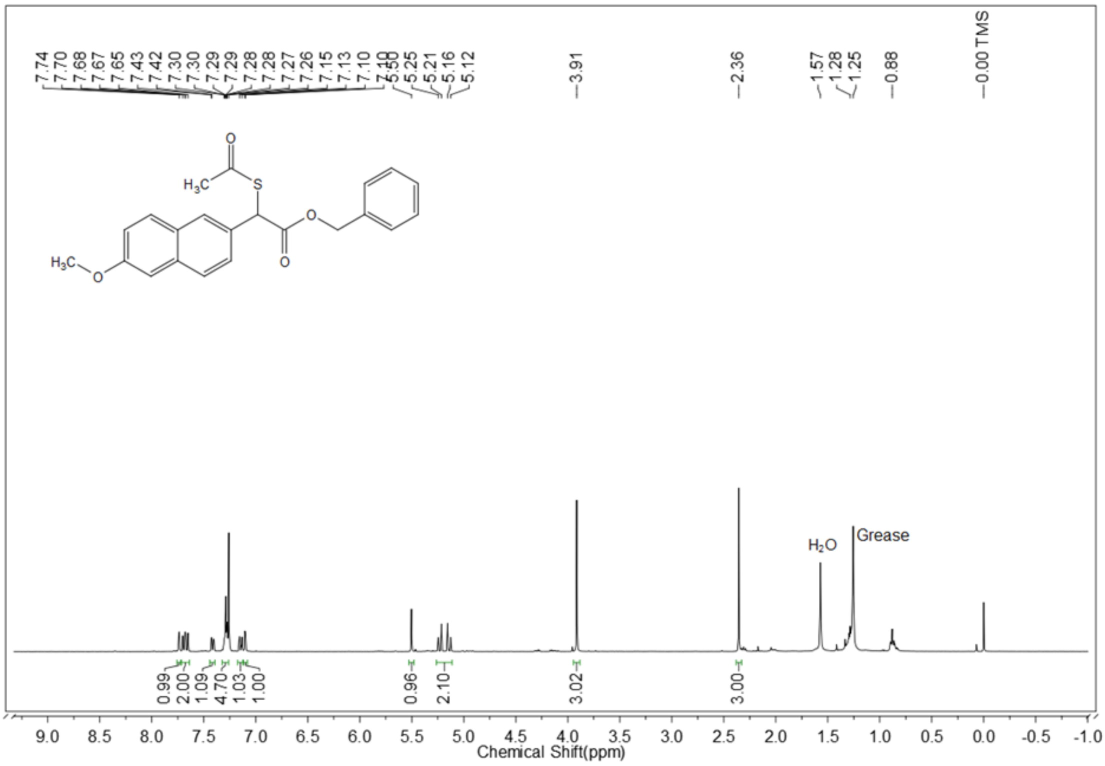

^{13}C NMR spectra of **7d**:

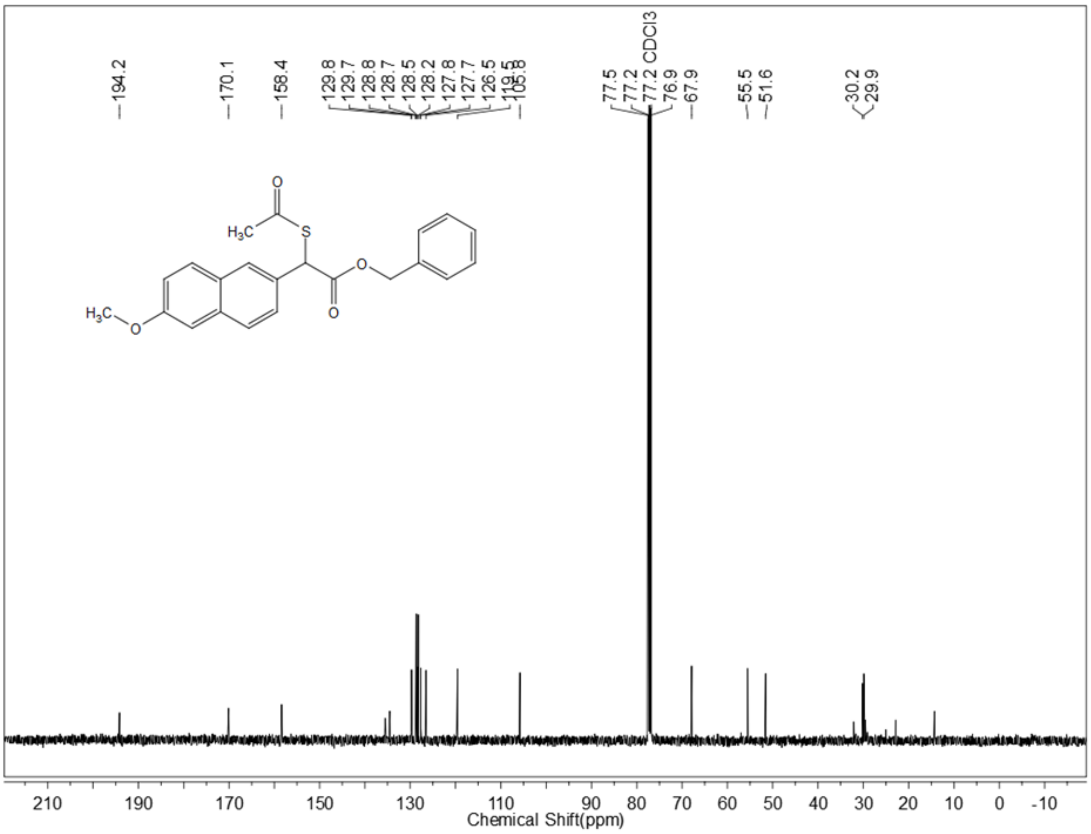

Supplement: SC-016-D4SC08525F-s001 [file SC-016-D4SC08525F-s001.pdf]
